# Compositional Engineering of NiSe_2_ Precatalysts with IrO_*x*_ for Controlled Reconstruction toward Improved Alkaline OER

**DOI:** 10.1021/acsami.5c15821

**Published:** 2025-11-23

**Authors:** Pâmella S. Rodrigues, Tatiana Priamushko, Moisés A. de Araújo, Gabriel C. da Silva, Serhiy Cherevko, Edson A. Ticianelli

**Affiliations:** † São Carlos Institute of Chemistry, 153988 University of São Paulo, Av. Trab. Sancarlense, 400, 13566-590 São Carlos, Brazil; ‡ Helmholtz-Institute Erlangen-Nürnberg for Renewable Energy (IET-2), 28334 Forschungszentrum Jülich GmbH, Cauerstr. 1, 91058 Erlangen, Germany; § Chemistry Department, 28120 Federal University of Viçosa, Av. Peter Henry Rolfs, 36570-900 Viçosa, Brazil

**Keywords:** Structural reconstruction, Nickel Selenide, Nickel Oxyhydroxide, Metal Transition Chalcogenide, Oxygen Evolution Reaction

## Abstract

The development of efficient and durable oxygen evolution reaction (OER) electrocatalysts remains a challenge in alkaline water electrolysis. Nickel-based materials are well explored as OER precatalysts due to their ability to undergo structural reconstruction into an active nickel oxyhydroxide phase (NiOOH) under anodic conditions. Interestingly, nickel-based chalcogenides, such as nickel selenides, also undergo reconstruction but form a more active surface compared to conventional nickel oxide catalysts, making them appealing for water electrolysis application in alkaline media. Here, we explore a strategy to modulate the chemical composition of nickel selenide by incorporating small amounts of iridium oxide (IrO_*x*_) nanoparticles. A series of catalysts with varying Ni:Ir ratio proportions were synthesized, and we demonstrate that IrO_*x*_ incorporation leads to a reduction in particle size, an increase in surface area, and a modification of surface composition, resulting in a lower OER overpotential. Online electrochemical dissolution measurements reveal that selenium acts as a sacrificial species during the structural reconstruction to form the NiOOH phase, while IrO_*x*_ suppresses excessive Se leaching. This behavior is rationalized using hard–soft acid–base theory, where the replacement of soft-base Se_2_^2–^ by hard-base oxygen species is driven by stronger interactions with Ni^3+^, a hard acid. The optimal composition (IrO_*x*_-NiSe_2_ with Ir atomic percentage of 2.4%) achieves a balance between accessible active sites and favorable intermediate adsorption. In contrast, higher IrO_*x*_ content led to performance decline due to site blocking, as predicted by Sabatier’s principle. This work highlights how compositional control and surface reconstruction can guide the design of OER precatalysts, while also deepening the understanding of oxidative transformation mechanisms in nickel chalcogenides, particularly selenides.

## Graphical abstract

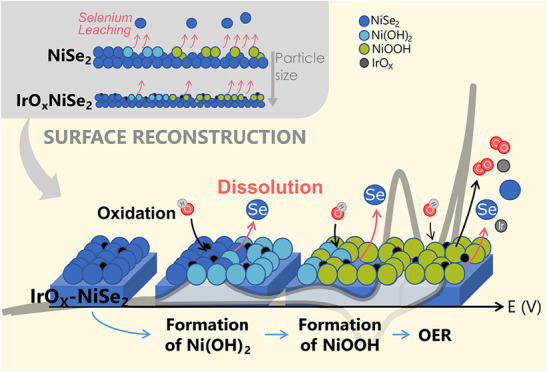

## 1 Introduction

Water electrolysis is a promising route for sustainable hydrogen production, offering a carbon-free alternative to conventional processes such as steam reforming of natural gas.^1^ The electrolysis process involves two half-cell reactions: the oxygen evolution reaction (OER) and the hydrogen evolution reaction (HER), which generate O_2_ and H_2_ gases, respectively.^1^ Hydrogen produced through electrolysis can be utilized in fuel cells or serve as a key feedstock for value-added chemicals, such as ammonia.^2^ Thermodynamically, water splitting requires a standard enthalpy of cell reaction of 285.8 kJ mol^–1^, corresponding to a thermoneutral cell voltage of 1.48 V_RHE_ at 25 °C. However, in practice, the sluggish kinetics of electrochemical reactions, especially OER, necessitate applying significantly higher voltages for electrolysis to occur at appreciable speeds.^3^

One strategy for conducting electrolysis reactions at lower overpotentials is to use active electrocatalysts such as noble metals like Pt and Ir. However, their scarcity contributes to high commercial costs. As a result, increasing attention is being given to exploring earth-abundant and cost-effective alternatives, such as transition metal-based catalysts.^4^ Among these, nickel-based compounds have shown considerable promise for OER electrolysis.^5^ Studies have shown that nickel-based catalysts, including nickel oxides,^6^ hydroxides,^7^ and chalcogenides,^8^ experience significant structural, morphological, compositional, and electronic transformations during the alkaline OER. In general, the oxidation process of Ni­(II) to Ni­(III) in the oxides is responsible for a structural reconstruction of the material, leading to the formation of oxide or oxyhydroxide species (NiOOH), the active phase responsible for catalyzing the OER.^9^

Nickel chalcogenide-based catalysts have emerged as a promising OER precatalyst due to their favorable reconstruction into the active NiOOH phase, which exhibits enhanced catalytic performance compared to NiOOH derived from conventional nickel oxides.^8,10^ This improved activity is often attributed to the formation of a surface phase-rich NiOOH layer, generated by the chalcogenide oxidation under anodic conditions.^11–13^ However, despite this benefit, their reconstruction is frequently accompanied by structural instability, particularly due to catalyst degradation.^13^ Recent approaches have focused on chemical modifications that incorporate secondary elements into metal chalcogenides (such as nitrogen,^14^ chromium,^14^ iron,^15^ and cobalt^16^) to modulate the electronic structure, stabilize the catalyst surface, and also optimize the catalytic performance. Similarly, incorporating small amounts of noble metals, such as iridium (Ir) and iridium oxide (IrO_*x*_), has demonstrated synergistic effects, enhancing both catalytic activity and stability. Specifically, previous studies on Ir–Ni systems have also demonstrated that Ir and IrO_*x*_ incorporation into nickel hydroxide/oxide matrices stabilizes the reconstructed NiOOH phase and tunes the adsorption strength of oxygenated intermediates, lowering the OER onset potential and accelerating reaction kinetics.^17,18^ These effects are particularly relevant for nickel chalcogenides, where IrO_*x*_ incorporation may not only improve the stability but also optimize the interfacial energetics between Ni and oxygen species. However, the optimal Ir/Ni ratio required to maximize OER performance in nickel chalcogenide-based material remains an open question.

In this work, we synthesized nickel selenide (NiSe_2_) precatalysts by introducing iridium oxide nanoparticles, varying the Ni:Ir ratio proportions via hydrothermal synthesis to investigate the influence of chemical modification on the catalytic performance and structural reconstruction of NiSe_2_. Our results reveal that IrO_*x*_ incorporation reduces particle size, increases the electrochemically active surface area (ECSA), and enhances the interaction with OER intermediates. We identified an optimal composition, balancing the chemical composition and enhancing the catalytic performance of NiSe_2_. Using advanced electrochemical techniques, a scanning flow cell (SFC) coupled with inductively coupled plasma mass spectrometry (ICP-MS),^19^ we confirmed that forming the NiOOH active stable phase is accompanied by Se anodic dissolution, and that IrO_*x*_ addition mitigates this process. These findings provide insights into the oxidative reconstruction of nickel chalcogenides, particularly nickel selenide.

## 2 Results

### 2.1 Physical and Chemical Characterization

NiSe_2_ modified with IrO_*x*_ nanoparticles was synthesized by the hydrothermal method, as described in the Section 5, with different molar ratios of Ni:Ir, i.e., 2:0 (pristine NiSe_2_), 2:0.1, 2:0.2, 2:0.4, and 2:0.8. The sample nomenclature adopted throughout the manuscript corresponds to the molar ratios used during synthesis. Elemental compositions were estimated by energy-dispersive X-ray spectroscopy (EDS) analysis (Table S1) and elemental mapping (Figure S2), confirming the presence of Ni, Se, Ir, and O in the synthesized IrO_*x*_-NiSe_2_ materials. The IrO_*x*_ content incorporated into the 2:0.1, 2:0.2, 2:0.4, and 2:0.8 samples were 1.2, 2.4, 5.9, and 9.2 atomic percent (at.%), respectively. The Se/Ni atomic percentage ratio of pristine NiSe_2_ was 1.92, which is very close to the expected stoichiometry (i.e., Se/Ni = 2). However, upon incorporating IrO_*x*_ nanoparticles, a decrease in the apparent Se/Ni ratio was observed (Se/Ni ratio varied between 1.48–1.66), representing ca. 23%–14% decrease in the Se/Ni ratio of the IrO_*x*_-NiSe_2_ compared to that of the pristine NiSe_2_, which is primarily due to a reduction in the measured selenium content (Table S1), and indicates that IrO_*x*_ incorporation may have affected the elemental composition in the bulk region of the catalysts. This reduction most likely arises from the formation of oxygenated nickel species (e.g., NiO or Ni­(OH)_2_) during the synthesis. Specifically, when the Ni and Se precursors are mixed with the hydrolyzed IrO_*x*_ solution (Figure S1). The basic environment may promote the formation of nickel-based oxide traces, which contribute additional Ni not bonded to Se.

The X-ray diffraction (XRD) patterns obtained for all Ni-containing catalysts, as shown in Figure 1a, reveal peaks corresponding to the formation of the cubic NiSe_2_ phase (Powder Diffraction File (PDF) no. 65–1843), regardless of the amount of IrO_*x*_ added. The XRD pattern for pristine IrO_*x*_ (PDF no. 15–870) shows a characteristic profile of amorphous material.^20,21^ The diffractogram displays two broad peaks in the 2θ ranges of 20–35° and 50–65°, which are close to the peaks of the IrO_2_ phase. The addition of IrO_*x*_ nanoparticles led to broader XRD peaks (full width at half-maximum (fwhm) varied from 0.30° to 0.42° at 2θ = 33.7°), indicating a decrease in the crystallinity degree and possibly an overlapping of the amorphous halo of IrO_*x*_ with the XRD peaks of NiSe_2_ (Figure 1a). The peak broadening also suggests reduced crystallite sizes. To verify this, the XRD patterns were analyzed using the Scherrer equation (eq 5), revealing crystallite sizes of approximately 5.4, 4.4, and 4.0 nm for the 2:0, 2:0.2, and 2:0.4 samples, respectively. These results confirm the progressive reduction in crystallite size, which agrees with the broadening of the XRD peaks. Another important aspect to note is the nature of the IrO_*x*_-NiSe_2_ material, which possibly behaves as a composite. The presence of IrO_*x*_ phase could not be detected in the IrO_*x*_-NiSe_2_ composite via XRD measurements owing to the amorphous nature of IrO_*x*_ (Figure 1a).

**Figure 1:**
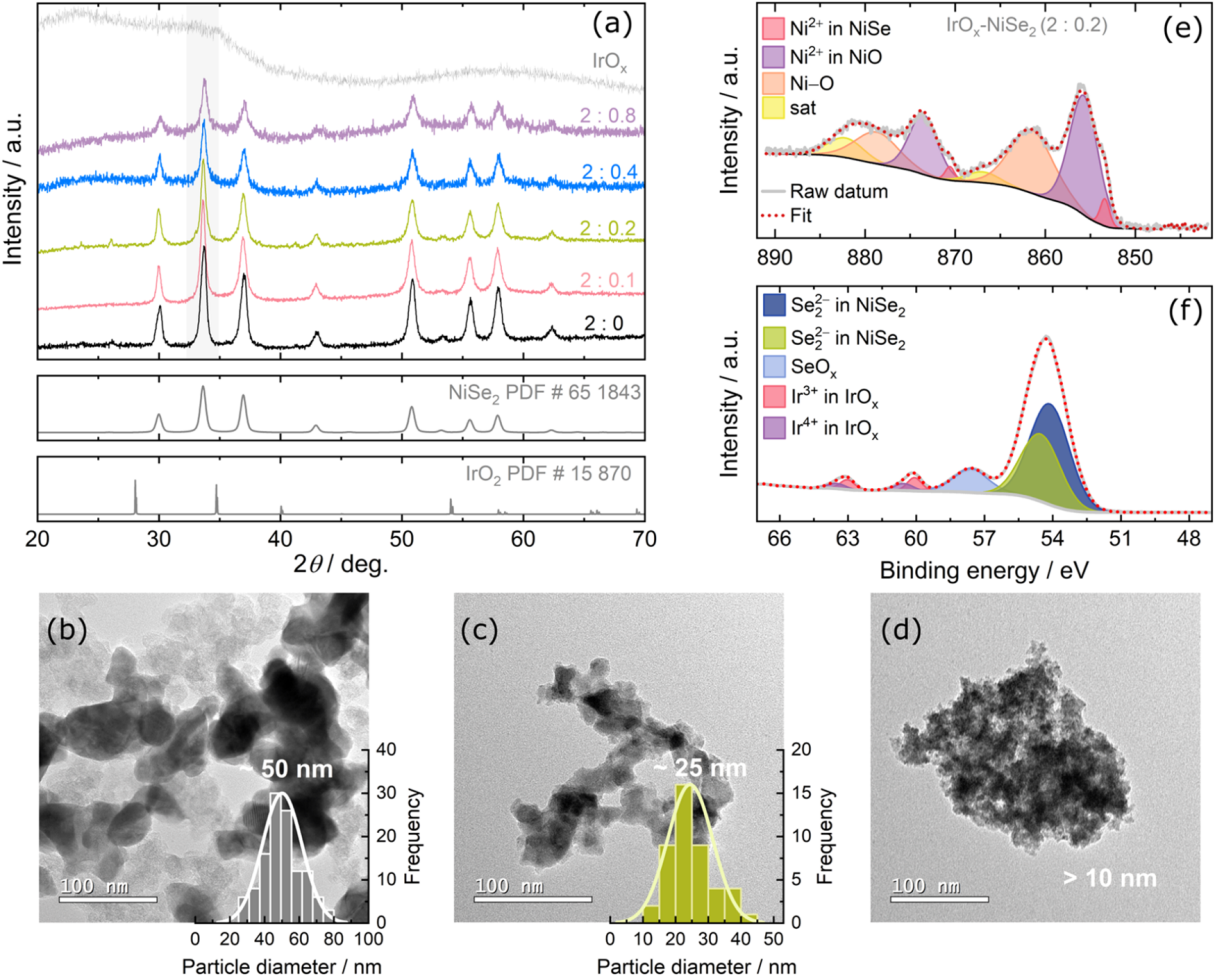
(a) XRD patterns of pristine NiSe_2_ and IrO_*x*_-NiSe_2_ with different molar ratios of Ni:Ir. TEM micrographs and their corresponding histograms of (b) pristine NiSe_2_ and IrO_*x*_-NiSe_2_ with molar ratios of Ni:Ir of (c) 2:0.2 and (d) 2:0.8. High-resolution XPS spectra of (e) Ni 2p and (f) Se 3d and Ir 4f core levels of IrO_*x*_-NiSe_2_ with a molar ratio of 2:0.2 (Ni:Ir).

Transmission electron microscopy (TEM) micrographs indicate that pristine NiSe_2_ has an average particle size of 50 nm (Figure 1b). The IrO_*x*_-NiSe_2_ catalyst with a Ni:Ir proportion of 2:0.2 has a size of 25 nm (Figure 1c), whereas the 2:0.8 proportion could not be accurately measured due to particles agglomeration (as also in Figures 1d and S3). It is important to note that the crystallite size derived from XRD refers to the size of diffracting domains. In contrast, the particle size observed by TEM refers to the physical dimensions of the entire particle, which may consist of multiple crystallites.^22^ Both crystalline and particle sizes decrease upon the presence of IrO_*x*_ content, indicating that IrO_*x*_ affects nucleation and growth processes during synthesis. The decrease of particle size may have also led to enlargement of the surface area of the catalysts, which is substantiated by the ECSA analyses as further discussed in Figure 4c.

The pristine NiSe_2_, IrO_*x*_ and IrO_*x*_-NiSe_2_ catalysts were further chemically characterized by X-ray photoelectron spectroscopy (XPS). XPS measurements were performed only for the IrO_*x*_-NiSe_2_ catalysts with (2:0.2), and (2:0.8) of Ni:Ir molar ratios since they represent a low and high degree of modification of the NiSe_2_ material, providing enough understanding of the chemical modification of the IrO_*x*_-NiSe_2_ materials. The fit parameters employed to model peaks and baseline of the XPS spectra of all samples are listed in Tables S2–S5. Considering initially the pristine NiSe_2_ (Figure S4a), the high-resolution XPS spectrum of Ni 2p shows three doublets with photoemission peaks at 853.0 (Ni 2p_3/2_) and 870.2 eV (Ni 2p_1/2_), 853.6 (Ni 2p_3/2_) and 871.3 eV (Ni 2p_1/2_), and 855.0 (Ni 2p_3/2_) and 873.0 eV (Ni 2p_1/2_), associated with Ni^2+^ in NiSe_2_,^23^ Ni^2+^ in NiO,^24^ and Ni–O bond,^23^ respectively. One also notices peaks at 860.1 and 877.5 eV ascribed to the satellite peaks.^23^ These assignments are consistent with the high-resolution Se 3d XPS spectrum of pristine NiSe_2_ (Figure S4d), which shows a doublet with peaks at 54.5 (Se 3d_5/2_) and 55.7 eV (Se 3d_3/2_) assigned to the Se_2_^2–^ in NiSe_2_.^23^ The additional peak at 58.4 eV is associated with SeO_*x*_.^23^

For the pristine IrO_*x*_, the high-resolution XPS spectrum of Ir 4f (Figure S5a) displays two doublets, one with peaks at 62.4 (Ir 4f_7/2_) and 65.3 eV (Ir 4f_5/2_) associated with the Ir^3+^ in IrO_*x*_, and the other one with peaks at 63.4 (Ir 4f_7/2_) and 66.3 eV (Ir 4f_5/2_) are assigned to the Ir^4+^ in IrO_*x*_.^20^ It is also noted peaks at 65.7, 62.9, and 61.9 eV ascribed to the satellite peaks.^25^ The high-resolution XPS spectrum of O 1s of pristine IrO_*x*_ (Figure S5b) features peaks at 530.6, 531.9, and 533.0 eV assigned to the lattice oxygen in the Ir–O–Ir bonding, Ir–OH bonding, and adsorbed water onto the material surface, respectively.^25,26^

Concerning the NiSe_2_ with IrO_*x*_ (Ni:Ir ratio of 2:0.2), as shown in Figure 1e, the high-resolution XPS spectrum of the Ni 2p core level displays a doublet with photoemission peaks at 869.7 (Ni 2p_1/2_) and 852.4 eV (Ni 2p_3/2_) assigned to Ni^2+^ in NiSe_2_.^23^ Two doublets were also noted, the first one at 854.8 (Ni 2p_3/2_) and 872.2 eV (Ni 2p_1/2_) attributed to the Ni–O bonding, and the second one at 859.6 (Ni 2p_3/2_) and 876.8 eV (Ni 2p_1/2_) are also assigned to Ni–O bonding possibly formed due to air exposure.^23,27^ The features at 862.7 and 880.3 eV are related to satellite peaks.^23,28^ The high-resolution XPS spectrum of Se 3d (Figure 1f) shows a doublet with deconvoluted peaks at 54.1 (Se 3d_5/2_) and 54.5 eV (Se 3d_3/2_) corresponding to Se_2_^2–^ in NiSe_2_,^23,28^ and a peak at 57.6 eV assigned to SeO_*x*_.^23^ The high-resolution XPS spectra of Ir 4f core level (Figure 1f) denote the presence of IrO_*x*_ in NiSe_2_ catalysts, as confirmed by the doublet with photoemission peaks at 60.6 eV (Ir 4f_7/2_), 63.5 eV (Ir 4f_5/2_) corresponding to Ir^4+^, and another doublet with peaks at 60.1 eV (Ir 4f_7/2_) and 63.0 eV (Ir 4f_5/2_) corresponding to Ir^3+^; both doublets are assigned to the Ir–O bondings.^20,21^ The high-resolution XPS spectra of IrO_*x*_-NiSe_2_ (Ni:Ir ratio of 2:0.8) feature similar peak profiles compared to that of the IrO_*x*_-NiSe_2_ (Ni:Ir ratio of 2:0.2). Peak assignments of the XPS spectra of the IrO_*x*_-NiSe_2_ (Ni:Ir ratio of 2:0.8), together with those of all other samples are summarized in Table S6. The high-resolution XPS of O 1s and C 1s are shown and discussed in Figure S4.

Based on this analysis, it is, therefore, possible to identify in all analyzed samples the peaks referring to the presence of NiSe_2_, nickel oxide and selenium oxide (SeO_*x*_) as already observed in the literature.^28^ Since XPS is a highly surface-sensitive technique (X-ray penetration depth of ca. 10 nm or less),^29^ the SeO_*x*_ and nickel oxide are certainly present at a minimal amount on the surface of the catalysts, as we did not identify these phases in the XRD data (cf. Figure 1a). This means that the bulky domain of the catalysts remains predominantly as NiSe_2_, while the surface oxide compounds may have formed during the synthesis, as previously discussed. Compared to the pristine NiSe_2_, increasing the IrO_*x*_ content resulted in the decrease of the Ni 2p peaks of the NiSe_2_ and an increase of the Ni 2p peaks of the nickel oxide (Figure S4a–c). This indicates that the formation of surface nickel oxides may have been favored for a higher amount of IrO_*x*_ incorporation in NiSe_2_. Furthermore, one notices a red shift of the Se 3d peaks for the IrO_*x*_-NiSe_2_ (shifts of ca. 0.7 and 0.3 eV for the Ni:Ir of 2:0.2 and 2:0.8, respectively) compared to that of the Se 3d peaks of the pristine NiSe_2_. A red shift of about 0.3 eV of Ni 2p peaks for the IrO_*x*_-NiSe_2_ (Ni:Ir ratio of 2:0.2) and a blue shift of ca. 0.8–0.4 eV for the IrO_*x*_-NiSe_2_ (Ni:Ir ratio of 2:0.8), are observed in comparison to those appearing for the pristine NiSe_2_. The Ir 4f peaks of the IrO_*x*_ also feature a red shift of ca. 2.1 and 1.6 eV for the Ni:Ir ratio of 2:0.2 and 2:0.8, respectively, in relation to Ir 4f peaks of the pristine IrO_*x*_. The red/blue shifts may be due to the electronic interaction between NiSe_2_ and IrO_*x*_ and possibly nickel oxide, as observed in other studies involving NiSe_2_ combined with other metal chalcogneides.^30–32^

Table S7 presents the surface elemental composition of the samples obtained by XPS. For the unmodified NiSe_2_ catalyst, the atomic percentages of nickel, selenium, and oxygen were 17.7%, 36.7%, and 6.80%, respectively. Upon incorporation of IrO_*x*_, a gradual reduction in surface nickel content and an increase in oxygen and iridium levels were observed. In this way, in the IrO_*x*_-NiSe_2_ sample with a Ni:Ir ratio of 2:0.2, the atomic composition reached 14.5% Ni, 41.7% Se, 3.1% Ir, and 12.2% O. For the 2:0.8 sample, the nickel content further decreased to 8.60%, while iridium and oxygen increased to 12.5% and 12.2%, respectively. These changes indicate that IrO_*x*_ incorporation alters the surface composition substantially, enriching the surface in oxygen-containing species.

A relevant point to consider is the contrast between the surface and bulk compositions, particularly regarding the nickel content. While XPS analysis reveals a marked decrease in surface Ni concentration with increasing IrO_*x*_ loading, EDS measurements indicate no changes in the bulk composition. Based on the XPS results, the Se/Ni ratios (Table S7) derived from XPS increase from 2.07 (pristine NiSe_2_) to 2.88 (Ni:Ir ratio of 2:0.2) and 3.65 (Ni:Ir ratio of 2:0.8), reflecting a depletion of nickel at the surface. In addition, the surface Ir/Ni atomic ratios (Table S7) calculated from the XPS indicate a progressive increase over increasing IrO_*x*_ content, from 0 (pristine NiSe_2_) to 0.21 and 1.45 for the 2:0.2 and the 2:0.8 samples, respectively, highlighting the progressive surface enrichment of Ir relative to Ni. These differences suggest that IrO_*x*_ phases tend to segregate at the catalyst’s surface, since bulk analyses by EDS show that the amount of Ni remains unchanged, leading to a surface-enriched oxide layer.

### 2.2 Structural Reconstruction of NiSe_2_ and IrO_*x*_-NiSe_2_ Precatalysts

In general, the redox process of Ni­(II) to Ni­(III) that occurs before the OER is responsible for a structural modification of the catalyst, leading to increased electronic conductivity and the formation of a more active surface layer.^33^ The NiSe_2_ precatalyst and IrO_*x*_-NiSe_2_ were initially analyzed by cyclic voltammetry (CV) to investigate the structural reconstruction during ten scan cycles of the voltammetry. The choice of 10 cycles was based on preliminary CV measurements, which showed that after ten cycles, the current density and peak positions stabilized, indicating that the surface modification had reached a steady state. Figure 2 presents the initial measurements of CVs using the RDE configuration in 1 mol L^–1^ KOH.

**Figure 2:**
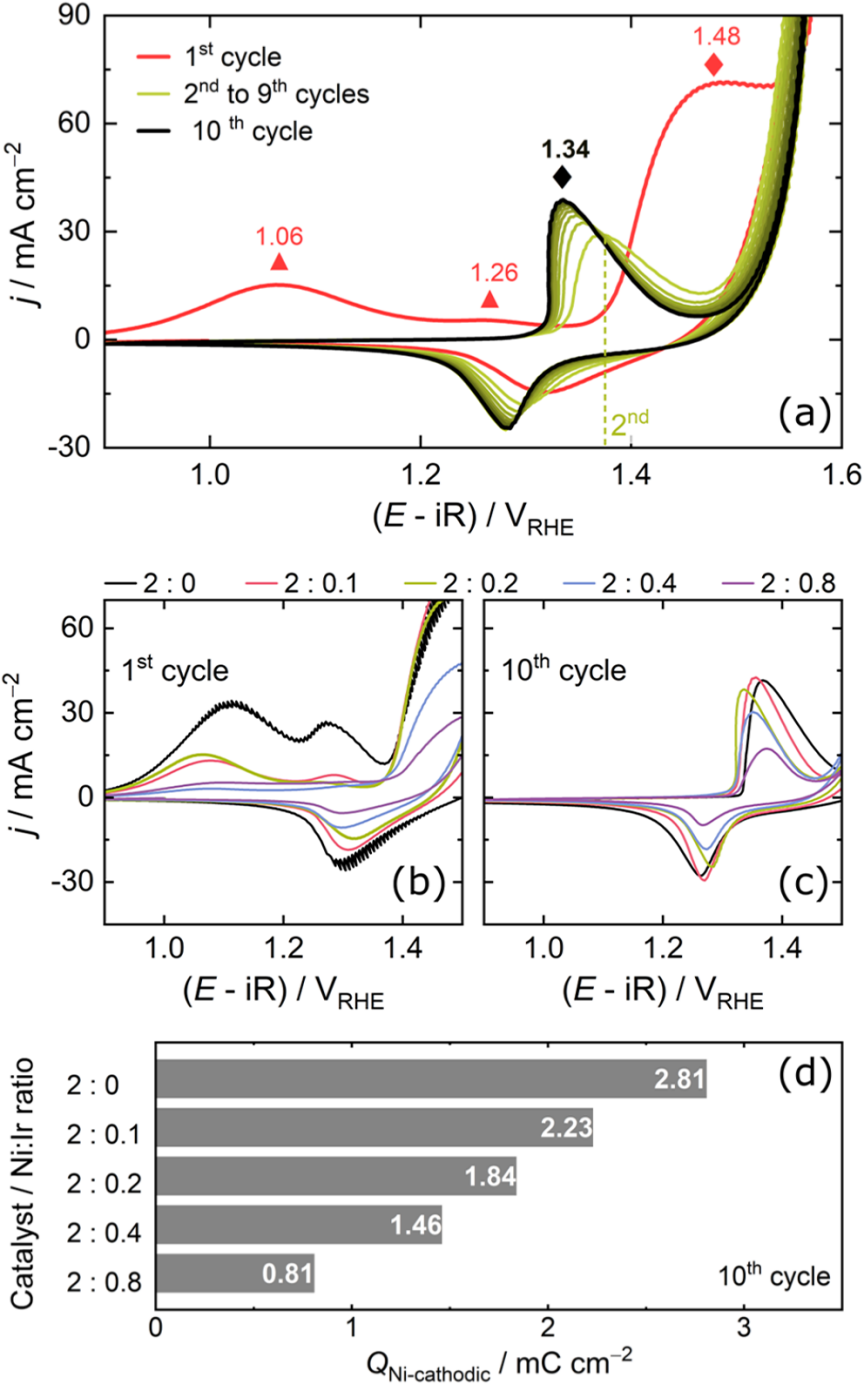
(a) Cyclic voltammograms at a scan rate of 100 mV s^–1^ of IrO_*x*_-NiSe_2_ (with Ni:Ir ratio of 2:0.2) with a highlight of the oxidation peaks of Se (▲) and Ni (⧫). Cyclic voltammograms at 100 mV s^–1^ for the (b) first and (c) tenth scan of pristine NiSe_2_ and IrO_*x*_-NiSe_2_ with different molar ratios of Ni:Ir. (d) Charge density of the nickel reduction peak obtained at the tenth scan of the CV. The electrolyte was an Ar-saturated solution of 1 mol L^–1^ KOH.

The first cycle reveals two oxidation processes with maximum current peaks at approximately 1.10 and 1.25 V_RHE_, as indicated in Figure 2a and for all samples in Figure S6, which may be associated with the oxidation of the selenium species.^34,35^ Then, the oxidation of the Ni^2+^ to Ni^3+^ species occurs at a higher potential just during the first cycle. These phenomena are attributed to the formation of the NiOOH active phase, which is responsible for catalyzing the OER.^9,36^ In the intermediate CV scans (second to fourth), a progressive shift in the Ni^2+^/Ni^3+^ redox peaks is observed for all the samples (Figure S6). For example, in the case of IrO_*x*_-NiSe_2_ with a Ni:Ir ratio of 2:0.2 (Figure 2a), the maximum potential of the first peak appears at 1.48 V_RHE_, while the second one occurs at 1.36 V_RHE_. From the fourth scan onward, no significant variation is detected, and the Ni redox process stabilizes around 1.34 V_RHE_. This stabilization suggests that the structural change induced by Se oxidation no longer affects the Ni redox process. We observed the same behavior for all catalysts (Figure S6). Based on this initial voltammetry analysis, it seems that ten cycles are sufficient to modify the structure of both pristine and IrO_*x*_-modified NiSe_2_. Furthermore, the anodic peak associated with selenium oxidation during the first CV scan becomes less pronounced after the incorporation of IrO_*x*_, mainly for the catalyst with higher loading (Figure 2b). We hypothesis that this behavior arises from the formation of additional oxide species on the surface due to the presence of IrO_*x*_ and surface nickel oxide, as evidenced by the increase in the atomic percentage of Ir (Table S7). The formation of oxide species on the NiSe_2_ surface is most likely nickel oxide (evidenced by higher intensity nickel oxide peaks, see Figure S4c), which may have hindered the direct oxidation of surface Se species during the initial CV scans, thus resulting in a decrease in the selenium oxidation peak (Figure 2b).

During the ten scans, the precatalysts formed a surface with a NiOOH phase, as further discussed in Section 2.3. As shown in Figure S6, the anodic current density associated with Ni oxidation exhibited a large wave in the first peak, given the difficulty in estimating the integrated charge density from the anodic curve in the first scan. The peaks corresponding to the Ni^3+^ to Ni^2+^ reduction (*Q*_Ni_) were integrated instead, as proposed by Batchellor and Boettcher,^37^ to estimate changes in the amount of electrochemically active Ni species on the surface during the reconstruction process. Figure S7 shows that, while the unmodified sample does not exhibit a significant increase in *Q*_Ni_, the IrO_*x*_-modified samples progressively increase it over successive cycles and then tend to approach stabilization (particularly in NiOOH form). The increase in *Q*_Ni_ is more pronounced in the samples modified with IrO_*x*_, likely due to the reduced particle size observed in the TEM images, which may enhance the accessible surface area and facilitate Ni activation.

When comparing the different materials after ten CV scans, cathodic *Q*_Ni_ (Figure 2c) decreases from 2.81 mC cm^–2^ for NiSe_2_ to 0.81 mC cm^–2^ for IrO_*x*_-NiSe_2_ with the highest IrO_*x*_ loading (Ni:Ir ratio of 2:0.8). A similar trend is observed for the anodic *Q*_Ni_, indicating that the incorporation of IrO_*x*_ reduces the number of electroactive Ni species available at the surface. Interestingly, the potential associated with Ni^2+^ to Ni^3+^ oxidation (*E*_Ni_) at the tenth scan (Figure 2c) does not follow a linear trend across the series (see Table S8): *E*_Ni_ initially decreases from 1.37 V_RHE_ (NiSe_2_) to 1.34 V_RHE_ (Ni:Ir ratio of 2:0.2) but then increases to 1.57 V_RHE_ at higher IrO_*x*_ loadings (Ni:Ir ratio of 8:0.8), suggesting that moderate IrO_*x*_ incorporation facilitates Ni oxidation by enhancing surface exposure and promoting the favorable formation of NiOOH phase. However, we suspect that while the incorporation of IrO_*x*_ initially enhances surface area exposure, its excessive incorporation reduces the number of electrochemically accessible Ni species at higher loadings. This result is supported by TEM and XPS analysis, which confirms a reduction in particle size and a lower atomic percentage of Ni on the surface, aligning with the electrochemical results.

To explore the impact of structural reconstruction on the materials, we analyzed the NiSe_2_ and IrO_*x*_-NiSe_2_ precatalysts using a SFC coupled to online ICP-MS.^19^ This advanced technique facilitates real-time analysis of dissolved species from the electrode surface during electrochemical measurements, offering insights into the catalytic processes. Based on the above-mentioned CV results, we developed the electrochemical protocol at a lower scan rate (CV scanned from 0.3 to 1.5 V_RHE_) that would allow us to follow the dissolution of Ni, Se, and Ir in the potential window of the redox processes. The scan rate was chosen to be 2 mV s^–1^ to separate possible overlapping dissolution peaks caused by the redox reactions. Figure 3a presents the electrochemical online dissolution of species during protocol at a lower scan rate for the pristine NiSe_2_ and IrO_*x*_-NiSe_2_ (Ni:Ir ratio of 2:0.2).

**Figure 3:**
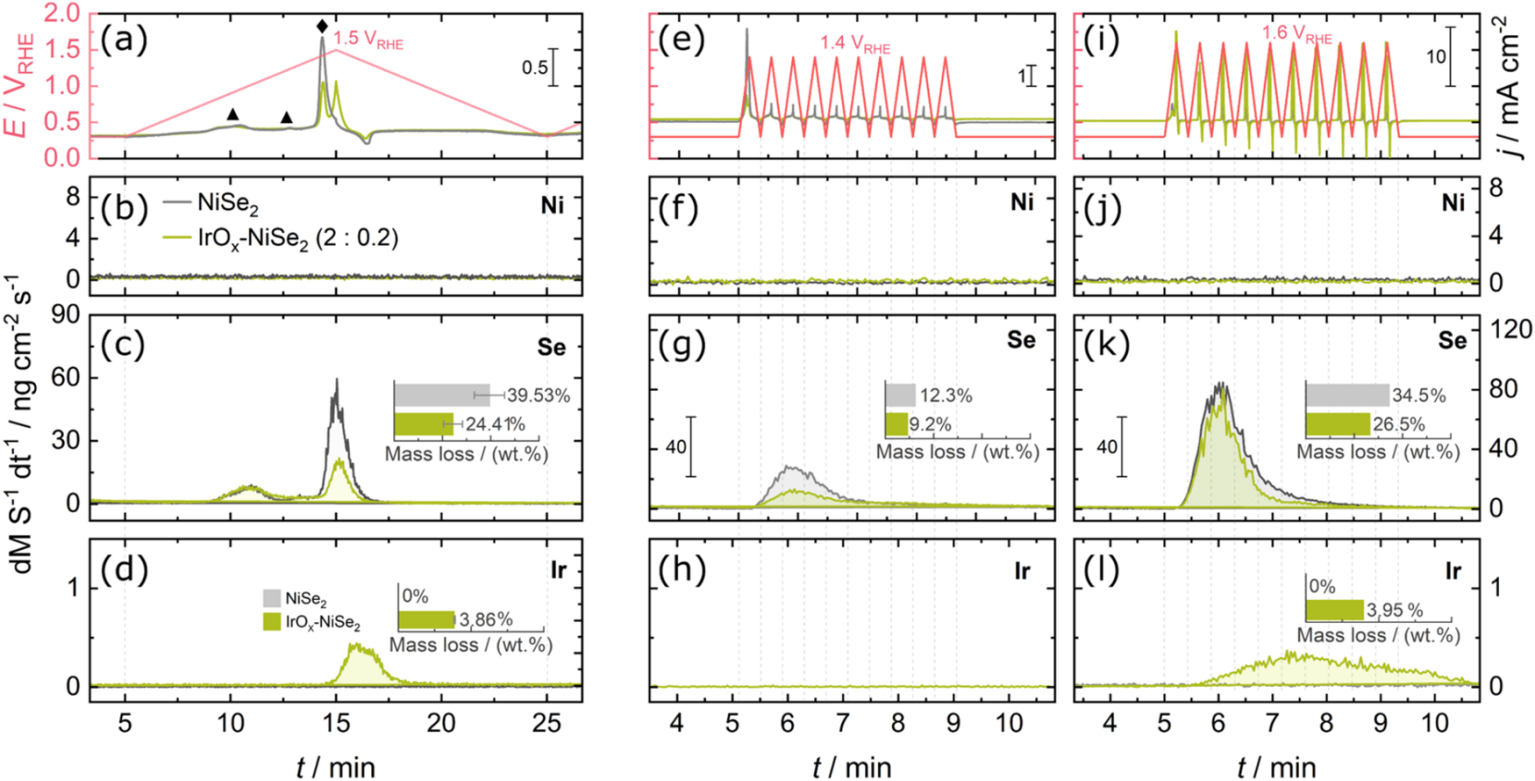
Formation of NiOOH during the (a) protocol at lower scan rate of NiSe_2_ pristine and IrO_*x*_-NiSe_2_ (Ni:Ir ratio of 2:0.2), accompanied by (b) Ni, (c) Se, and (d) Ir dissolution profiles at 2 mV s^–1^. Dissolution of (f, j) Ni, (g, k) Se, and (h, l) Ir during (e) *Protocol II* and (i) *Protocol III*, respectively, at 100 mV s^–1^. The electrolyte was an Ar-saturated solution of 0.1 mol L^–1^ KOH.

The Ni dissolution profile (Figure 3b) shows no detectable signal, indicating the stability of the NiOOH phase, consistent with the Pourbaix diagram (Figure S8). In contrast, the Se dissolution profile (Figure 3c) reveals two distinct events. Initially, Se_2_^2–^ species undergo oxidation and dissolution starting at 0.77 V_RHE_. At higher potentials, at 1.39 V_RHE_ for pristine NiSe_2_ and 1.42 V_RHE_ for IrO_*x*_-NiSe_2_, further Se dissolution occurs. According to previous studies,^13,38^ the initial reconstruction of NiSe_2_ involves the formation of Ni­(OH)_2_, followed by oxidation of Ni^2+^ to Ni^3+^, leading to the formation of the active NiOOH phase. Our results suggest that the surface reconstruction of NiSe_2_ begins at 0.77 V_RHE_ via the oxidation and dissolution of Se species into soluble Se oxyanions. At potentials above 1.39 V_RHE_, residual Se species in the NiSe_2_ are also leached, concomitant with formation and growth of NiOOH species. This behavior is entirely consistent with previous studies,^13^ which have directly detected the formation of selenite (SeO_3_^2–^) and selenate (SeO_4_^2–^) species as the predominant dissolved products under alkaline OER conditions,^13^ as also suggested by Se Pourbaix diagram (Figure S9).

IrO_*x*_ species also remain stable during the oxidation of Ni^2+^ (Figure 3c) and only dissolve under more oxidizing conditions, starting around 1.44 V_RHE_, which is probably associated with the OER process, indicating that Se undergoes preferential and earlier oxidation compared to Ir at higher oxidation conditions. While IrO_*x*_ incorporation does not entirely prevent Se oxidation and leach, this process is hindered upon IrO_*x*_ presence. To quantify the amount of dissolved species, the dissolution profiles were integrated, and the results of total dissolution are presented in Figure S10. For pristine NiSe_2_, 6.93 μg cm^–2^ of Se was dissolved during the first cycle, corresponding to approximately 39% of the initial Se content (17.5 μg cm^–2^). In contrast, for IrO_*x*_-NiSe_2_, the dissolved Se reaches 3.70 μg cm^–2^, which represents 24% of the initial Se mass (15.1 μg cm^–2^). These results indicate that the incorporation of IrO_*x*_ can suppress the anodic dissolution of selenium, in agreement with the electrochemical behavior observed in Figure 2b.

To further investigate how dissolution evolves during structural reconstruction under CVs cycling, we performed additional measurements at faster scan rates (100 mV s^–1^) using different potential cycling. The protocols were designed based on the previous findings (Figure 2), with the potential ranges selected to capture different stages of the nickel redox process: before Ni oxidation (*Protocol I*, 0.3–1.2 V_RHE_, Figure S11), during Ni oxidation (*Protocol II*, 0.3–1.4 V_RHE_, Figure 3e), and at higher potential (*Protocol III*, 0.3–1.6 V_RHE_, Figure 3i). The dissolution profile was also integrated, and Figure S12 shows the total dissolution in each protocol.

The Ni dissolution profiles (Figures S11b and 3f,j) indicate that Ni remains stable during all stages of the nickel redox process, while Se undergoes continuous anodic dissolution (Figures S11c and 3g,k). At this faster scan rate, dissolution becomes cumulative by limiting the time for surface transformations and mass transport, precluding the distinction of individual dissolution peaks compared to those under slower conditions (Figure 3a). Although all protocols use the same scan rate, the extended potential range in *Protocol III* appears to be the dominant factor influencing Se dissolution. The total Se dissolution (Figure S12) is lower during the initial stages of reconstruction (*Protocol I* and *II)*; however, a higher mass loss of selenium is observed on the extended potential range (*Protocol III*).

Furthermore, the dissolution profiles in Figure 3k,l show that Se oxidizes and dissolves preferentially over IrO_*x*_, consistent with previous observations (Figure 3d, at a lower scan rate). Notably, the dissolution behavior of IrO_*x*_ also differs between protocols. In *Protocols I* and *II*, it confirms no IrO_*x*_ dissolution before and during the NiSe_2_ oxidation, whereas in the third protocol IrO_*x*_ dissolves (Figure 3l). Even in this case, determining the dissolution onset becomes uncertain. As already mentioned, the profile highlights that IrO_*x*_ dissolution can also be triggered by higher potential, possibly during the OER process, as previously confirmed in other work.^39^ These results reinforce that the higher potential not only intensifies Se-based specie dissolution but also contributes to triggering the IrO_*x*_ dissolution.

### 2.3 Electrochemical Characterization and OER Performance

The activity of the precatalysts (i.e., NiSe_2_) toward the OER was evaluated by linear sweep voltammetry (LSV) after ten CV scans (Figure S6) using RDE configuration in 1 mol L^–1^ KOH, as shown in Figure 4a. The catalysts with the highest OER activity are characterized by a lower onset potential (*E*_onset_) for the water oxidation reaction, after which the anodic current density values rise continuously for increasing electrode potentials.^40^ The *E*_onset_ was estimated by the tangent’s method,^41^ as indicated in Figure S13, and the values are presented in Table S8. Figure 4b shows that the *E*_onset_ changes from the lowest to the highest depending on the catalyst composition as follows: (2:0.8) > (2:0.4) ≥ IrO_*x*_ > (2:0.2) ≥ (2:0.1) > NiSe_2_. Regarding the overpotential to achieve a current density of 10 mA cm^–2^ (η_10_) for the OER (Table S8), the IrO_*x*_-NiSe_2_ features a minimum value of 279 mV_RHE_ for the Ni:Ir ratio of 2:0.4, which is lower compared to that of the pristine NiSe_2_ (η_10_ of 302 mV_RHE_). This result indicates that modifying NiSe_2_ with IrO_*x*_ results in improved OER performance.

**Figure 4:**
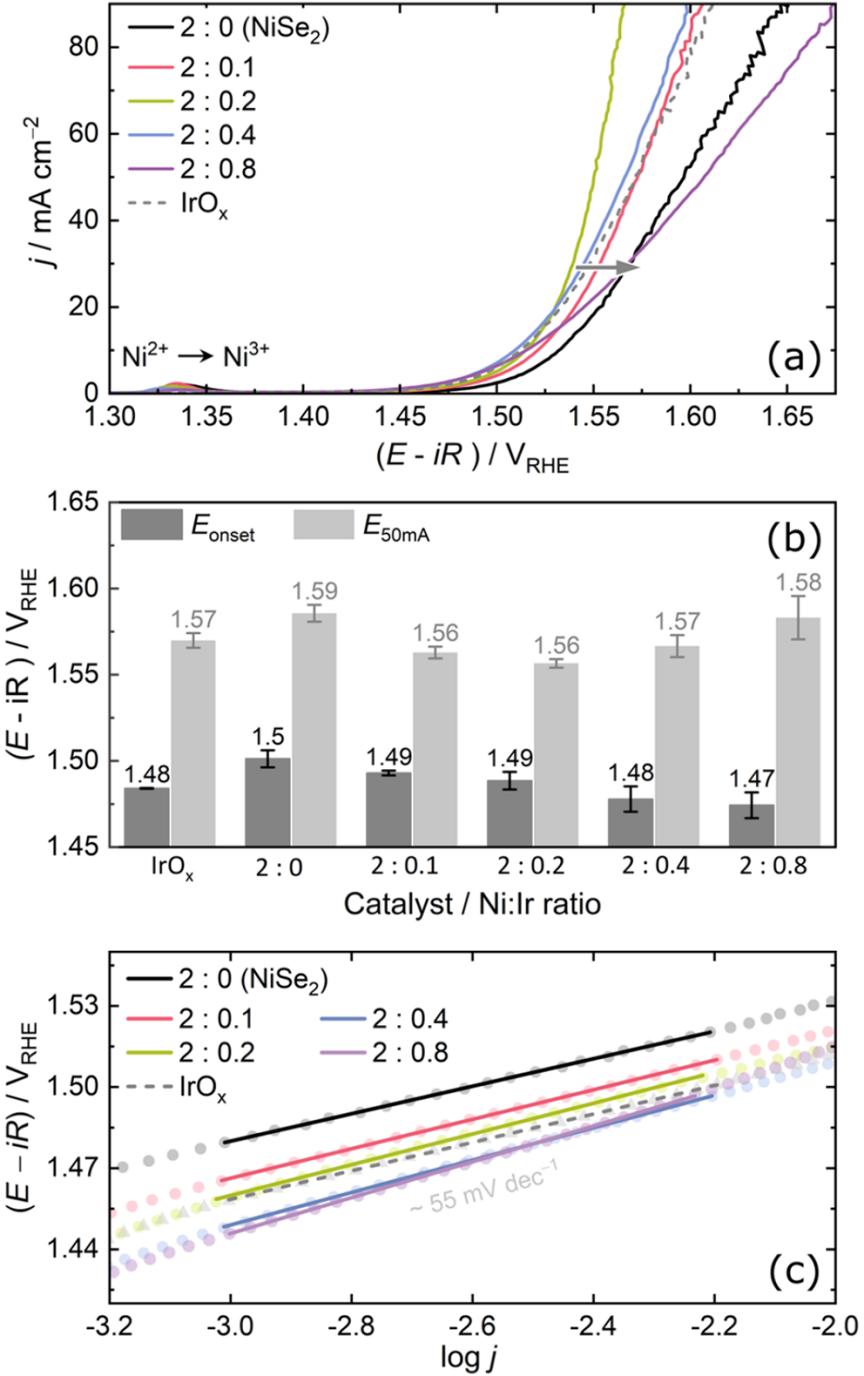
(a) LSV curves at a scan rate of 1 mV s^–1^ and (b) OER *E*_onset_ and *E* at 50 mA cm^–2^ (*E*_50 mA_) of NiSe_2_, IrO_*x*_, and IrO_*x*_-NiSe_2_ with different molar ratios of Ni:Ir. (c) Tafel plot obtained from the LSV curves. The electrolyte was an Ar-saturated solution of 1 mol L^–1^ KOH.

In addition, the complementary analysis of dissolution species during the LSV scan for NiSe_2_ and IrO_*x*_-NiSe_2_ (2:0.2) in 0.1 mol L^–1^ KOH after the *Protocol III*, as shown in Figure S14, helps contextualize the electrochemical behavior related to the OER process. After the precatalyst reconstruction (*Protocol III*), the onset of Se and Ir dissolution occurs at approximately 1.58 V_RHE_, which is at a higher potential than the OER *E*_onset_ (Table S8). Therefore, these results indicate that dissolution is not strictly concurrent with OER *E*_onset_. This shift in the dissolution onset potential from 1.39 and 1.44 V_RHE_ (for Se and Ir, respectively, as discussed previously) to 1.58 V_RHE_ after cycling suggests that surface reconstruction enhances surface stability, likely due to the formation of a stable passive nickel oxide layer, which delays the leaching of Se and Ir. However, at higher potentials, the oxidation and subsequent dissolution of these elements become unavoidable due to the enhanced charge transfer, which increases their interaction with OH^–^ and promotes catalyst degradation.

The polarization data corrected by the ohmic drop (Table S8) were plotted as Tafel diagrams (*E* vs log *j*), and the results are shown in Figure 4c. The Tafel slopes range from 53 to 70 mV dec^–1^, depending on the material composition (Table S8). Compared to the unmodified material (2:0), which displays a slope of 53 mV dec^–1^, the slope increases progressively with IrO_*x*_ incorporation, reaching 70 mV dec^–1^ for the 2:0.8 sample, an increase of approximately 32%. These variations in Tafel slopes may offer insights into the OER mechanism. However, in this case, ICP-MS data confirms the dissolution of Se species during the OER. This dissolution is associated with the continuous oxidation of NiSe_2_, which releases new nickel-oxidized sites and forms new active phases for O_2_ evolution. As a result, the measured Tafel slopes do not accurately reflect the intrinsic OER kinetics, limiting mechanistic interpretation.

According to the literature, the OER mechanism involves a multistep process with complex intermediates and a four-electron transfer per oxygen molecule. In alkaline media, most proposed mechanisms begin with the adsorption of hydroxide ions on the catalyst surface, followed by successive electron transfers.^8,42^ Usually, the increase in Tafel slope^43^ suggests a shift in the mechanistic pathways or rate-determining steps, often due to changes in surface coverage or other factors influencing the electron transfer steps, as noted in the literature.^43,44^ Our results align with this understanding, suggesting that while IrO_*x*_ incorporation facilitates the initial adsorption of OER intermediates,^17,45^ reflected in a decrease in *E*_onset_, it may also introduce surface changes that influence the subsequent electron transfer steps, leading to a higher Tafel slope.

Electrochemical impedance spectroscopy (EIS) was employed to probe the charge transfer processes of NiSe_2_, IrO_*x*_, and IrO_*x*_-NiSe_2_ catalysts. For pristine NiSe_2_ and IrO_*x*_ (Figure S15a,b), the complex-plane impedance and Bode diagrams were fitted using a classical Randles-type circuit (Figure S15c), comprised of a solution resistance (*R*_s_), charge transfer resistance (*R*_ct_), and a constant phase element associated with the double-layer capacitance (CPE_d_).^46^ In contrast, IrO_*x*_-NiSe_2_ (Figures S15d,e) could not be adequately modeled by a single *RC* element. Instead, the data were better described by the Armstrong-Henderson equivalent circuit (Figure S15f), as applied for metal transition-based electrodes,^47,48^ where the additional *RC* (i.e., *R*_ads._ and CPE_ads._) component is attributed to the adsorption of oxygenated intermediates on the catalyst surface during OER.^47,48^

The fitting parameters (Table S9) indicate a progressive increase in the values of *R*_ads_ with increasing IrO_*x*_ loading in NiSe_2_ and this may occur owing to the site-blocking effect. In other words, a higher loading of IrO_*x*_ has resulted in segregation of IrO_*x*_ on NiSe_2_ surface (as discussed in the characterization results), and the segregated phases may cover the NiOOH active sites for the OH^–^ adsorption. On the other hand, impedance shows a decrease in *R*_ct_ upon IrO_*x*_ incorporation, dropping from 47.02 Ω cm^2^ for pristine NiSe_2_ to values as low as 4.56 Ω cm^2^ for the IrO_*x*_-NiSe_2_ (2:0.8), highlighting the beneficial role of IrO_*x*_ in accelerating charge transfer kinetics. This result is consistent with the enhanced OER observed in the results shown in Figure 4b.

Additional evidence about the effect of IrO_*x*_ on the electrocatalytic reaction rates was achieved via the time constant (τ) associated with the charge transfer step, calculated from eq S5. As listed in Table S9, there was a gradual decrease in τ_ct_ for the modified catalysts, from 1.90 (pristine NiSe_2_) to 0.024 s (Ni:Ir ratio of 2:0.8), suggesting that charge transfer is faster for the catalyst modified with IrO_*x*_. This result also implies that modifying NiSe_2_ with IrO_*x*_ improves the electrocatalysis of the OER, which explains the shift in the *E*_onset_ toward less positive values, as observed in Table S8, and rationalizes the enhanced OER performance of IrO_*x*_-NiSe_2_. On the other hand, the τ_ads._ increased over increasing IrO_*x*_ content and this increase may be linked to the site-blocking effect, supporting our hypothesis mentioned above. Also, the results indicate that the dynamics of the adsorption–desorption process become slower, possibly due to a greater interaction of the OER intermediate species with the electrode.

Considering the equivalent circuit given in Figure S15f, in the conditions of the DC experiments (polarization curves, Figure 4a), the total charge transfer resistances (*R*_total_) calculated (*R*_total_ = *R*_ads_ + *R*_ct_) from the data in Table S9 evidence the following increment of catalyst activities (2:0.8) > (2:0.4) ≥ (2:0.2) > IrO_*x*_ > (2:0.1) > NiSe_2_ in the electrode potential of the impedance measurements (1.47 V_RHE_). This sequence is in quite good agreement with the current density magnitudes measured at 1.47 V_RHE_ in the polarization curves (Figure 4a).

Additionally, the total ECSA of the electrodes was assessed via their double-layer capacitance density (*C*_d_), as *C*_d_ and ECSA are directly proportional.^49^ It is worth noting that we did not perform the structural reconstruction of the catalysts (i.e., running the 10 CVs) prior to the *C*_d_ or ECSA analysis. The *C*_d_ was estimated by recording CVs in a nonfaradaic potential range (0.629–0.682 V_RHE_) at different scan rates (100–300 mV s^–1^), as shown in Figure S16. The capacitive current densities were calculated from the difference between the anodic and cathodic currents at 0.66 V_RHE_. The estimated *C*_d_ value corresponded to the slope of the linear equation of the (*j*_a_ – *j*_c_)/2 vs υ plots.^50^ As displayed in Figure S16f, the presence of IrO_*x*_ with a Ni:Ir ratio up to 2:0.2 resulted in a 3-fold increase of the *C*_d_ relative to that of the pristine NiSe_2_ (0.162 mF cm^–2^), which means enlargement of the total ECSA. In summary, this result denotes a volcano-type trend for the *C*_d_ values as a function of the increase of the Ir/Ni ratio, similar to what was observed for the performance of the OER potential above 1.55 V_RHE_. This result is supported by the TEM analyses that indicate a higher surface area of the IrO_*x*_-NiSe_2_ (2:0.2) material owing to the smaller particle size (Figure 1c,d).

Turnover frequency (TOF) values were calculated and normalized by the amount of electrochemically active NiOOH species to compare further the intrinsic activity of the catalysts (calculation details are found in the Supporting Information). As shown in Figure S17, at lower potentials (e.g., 1.47 V_RHE_), the TOF values increase from 0.22 s^–1^ for NiSe_2_ to 0.72 s^–1^ for IrO_*x*_-NiSe_2_ with the highest Ni:Ir proportion (2:0.8). This trend corroborates the improvement in activity observed by the *E*_onset_ values and can be attributed to optimized adsorption strength of OER intermediates upon IrO_*x*_ incorporation.

In addition, the TOF values increase with the applied potential for all catalysts, although this increase in terms of IrO_*x*_ content follows a nonlinear correlation. In fact, at potentials of 1.55 and 1.85 V_RHE_, the TOF values increase reaching a maximum for the catalyst with a 2:0.2 Ni:Ir ratio that presents the highest intrinsic activity (TOF = 18.6 s^–1^). Compared to a 2:0.2 Ni:Ir ratio, the 2:0.4 ratio catalyst exhibits a decrease in frequencies (TOF = 12.3 s^–1^), which subsequently (2:0.8 Ni:Ir ratio, TOF = 11.9 s^–1^), remains essentially constant. This behavior over increasing IrO_*x*_ loading is possibly due to stronger interaction of the OER intermediates with the catalyst surface,^51,52^ which hinders the adsorption–desorption dynamics, supporting thus the EIS results (τ_ads._) as mentioned before. This interpretation is also consistent with the lower current density observed in the LSV curves at higher *E*.

### 2.4 Catalyst Long-Term Stability

Accelerated degradation tests (AST) were conducted through CV scans to investigate the long-term stability of the catalysts. The protocol involved the use of the RDE system and running 500 CVs from 0.3 to 1.5 V_RHE_ at 100 mV s^–1^. Figure S18 shows the results for cycle numbers first and 500th, and LSV scan after 10th and 500th scans for NiSe_2_ and IrO_*x*_-NiSe_2_ (Ni:Ir ratio of 2:0.2). These results indicate a slight decrease in OER activity, suggesting a possible increase in the resistivity of the catalyst layer. Consistently, in both cases (pristine and modified NiSe_2_), there were no significant changes in the *E*_onset_ for the OER, evidence that the active NiOOH phase remains unchanged. In addition, there is an increase in the intensity of the NiOOH formation rate, but this process is shifted to higher potential, as indicated in Figure S18.

Before and after the AST test, the catalyst layer was analyzed by EDS and by identical location transmission electron microscopy (IL-TEM). Figure 5a,b shows the chemical composition of NiSe_2_ and IrO_*x*_-NiSe_2_ (Ni:Ir ratio of 2:0.2), before and after the AST test. The EDS analysis confirms the complete removal of selenium after the degradation test, caused by the oxidation of Se_2_^2–^ and Ni^2+^, indicating that the structural transformation of NiSe_2_ into the NiOOH active phase is not limited to the surface, but the catalyst is completely oxidized. The IL-TEM micrographs of the IrO_x_–NiSe_2_ (Ni:Ir of 2:0.2) catalyst before and after the AST (Figure 5c,d) evidence a depletion of particles in the analyzed region and the contraction of the existing clusters. This particle contraction phenomenon is a standard process in the degradation of nanoparticles, caused by a coalescence process in which the particle clusters reorganize themselves to form more compact structures with a lower overall surface area.^53,54^

**Figure 5:**
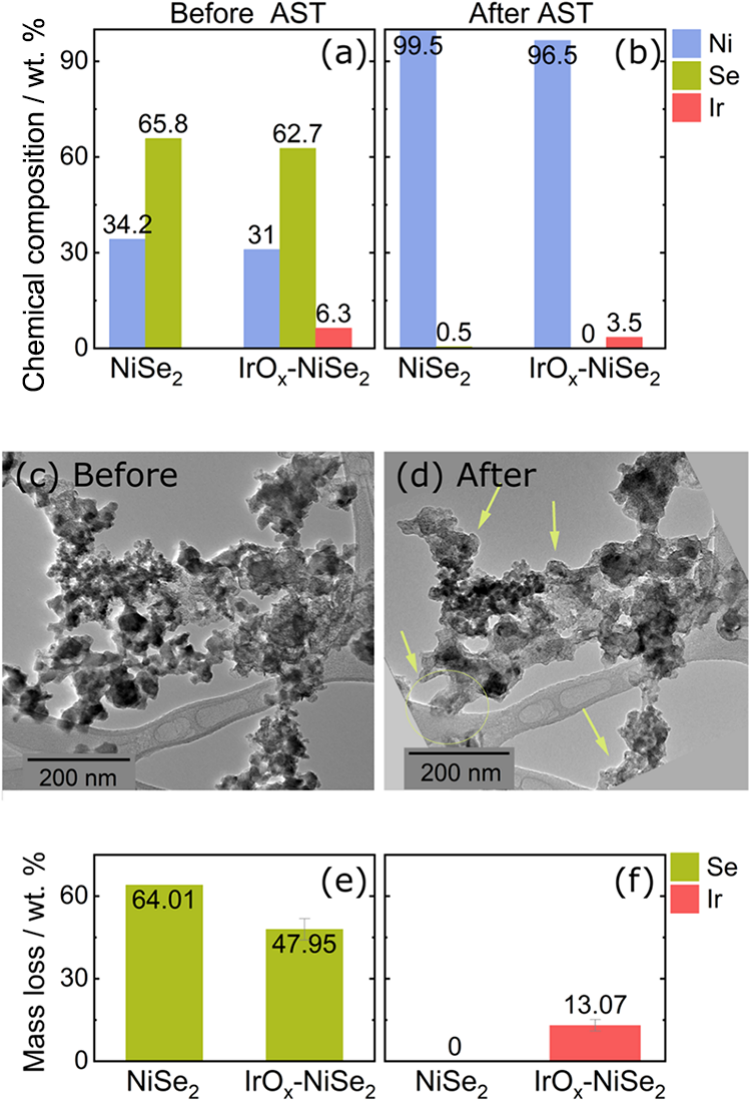
Chemical composition in terms of weigh percentage from the EDS analysis of (a) pristine NiSe_2_ and (b) IrO_*x*_-NiSe_2_ (Ni:Ir ratio of 2:0.2) before and after the AST. IL-TEM micrographs obtained from IrO_*x*_-NiSe_2_ (Ni:Ir ratio of 2:0.2) catalyst (c) before and (d) after AST. The electrolyte used for EDS and IL-TEM analysis was an Ar-saturated solution of 1 mol L^–1^ KOH. Mass loss of (e) Se and (f) Ir determined by ICP-MS during AST in Ar-saturated solution of 0.1 mol L^–1^ KOH for pristine NiSe_2_ and IrO_*x*_-NiSe_2_.

ICP-MS also analyzed the species dissolution during the AST in 0.1 mol L^–1^ KOH. Figure S19 shows the dissolution profiles of Ni, Se, and Ir during the AST measurements. The results confirm that the Ni content remains stable over prolonged cycling, as no Ni dissolution is detected (Figure S19a). In contrast, Se and Ir undergo substantial dissolution, especially within the first 10 cycles, and continue to dissolve gradually eventually decreasing below the detection limit (Figure S19b,c). The dissolution signals were fully integrated over the entire AST, the total Se and Ir are shown in Figure S20 and their respective mass loss in Figure 5e,f. The total Se loss from pristine NiSe_2_ reaches 11.2 μg cm^–2^, corresponding to 64% of the initial Se content as indicated in Figure 5e. For the IrO_*x*_-NiSe_2_, Se dissolution is reduced to 7.85 μg cm^–2^ (mass loss 47%), while Ir loss reaches 0.20 μg cm^–2^, accounting for 13% (Figure 5f). These findings indicate that although IrO_*x*_ is not fully stable under prolonged OER conditions, its incorporation moderates the dissolution of selenium during the oxidative cycles. Rather than preventing degradation, IrO_*x*_ appears to mitigate Se loss to some extent. Nonetheless, this attenuation does not imply an enhancement in long-term catalyst stability. Given the observed compositional changes from EDS analysis after the AST protocol in 1 mol L^–1^ KOH, it is likely that harsher alkaline environments or extended cycling would result in complete oxidation of the catalyst.

In addition to the AST CV-based experiments, the long-term stability of pristine NiSe_2_ and IrO_*x*_-NiSe_2_ catalysts were performed via chronopotentiometry at 10 mA cm^–2^ in 1 mol L^–1^ KOH, aiming to evaluate the catalysts under practical operating conditions of alkaline electrolyzers. The stability measurements were performed after the structural reconstruction protocol (i.e., after 10 CV scans). As shown in Figure S21, pristine NiSe_2_ exhibits an increase in potential during the initial 2 h of the experiment, likely due to additional structural reconstruction of NiSe_2_ to NiOOH. Moreover, one notices a sharp rise in potential after 8 h of continuous running experiment, which is associated with the detachment of the catalyst. For IrO_*x*_-NiSe_2_ (Ni:Ir ratio of 2:0.2), the potential slightly varied over 10 h of continuous electrolysis, and no catalyst detachment was observed during the experiment. Regarding the overpotential variation at 10 mA cm^–2^ (Δη_10 mA_) during 8 h of stability experiment (Figure S21), Δη_10mA_ values of 162 and 64 mV_RHE_ were achieved for pristine NiSe_2_ and IrO_*x*_-NiSe_2_ (Ni:Ir ratio of 2:0.2), respectively. This result indicates that neither catalysts are not fully stable under prolonged electrolysis, however, the NiSe_2_ catalyst modified with IrO_*x*_ shows an improvement in stability compared to that of the pristine NiSe_2_.

## 3 Discussion

The NiSe_2_-based materials were synthesized with varying molar ratios of Ni:Ir via introducing IrO_*x*_ nanoparticles, which influences particle nucleation and leads to a decrease in average particle size. A summary of the characterization results revealed that the incorporation of IrO_*x*_ alters the structural and surface properties of NiSe_2_ and reveals the formation of a surface with a decrease in Ni content. These modifications influence the structural reconstruction and enhance the OER activity behavior of NiSe_2_.

These materials act as precatalysts for alkaline OER, undergoing structural transformation under electrochemical conditions. The oxidation of selenium species has been previously proposed in studies on chalcogenide-based electrocatalysts based on the physical characterization of selenium on the catalyst surface.^55–57^ However, this is the first study to directly confirm the electrochemical dissolution of selenium via online detection using ICP-MS analysis. Our results confirm that Se_2_^2–^ species are selectively oxidized to higher state valences and leached from the structure during the activation process, as a sacrificial chalcogenide. These Se species dissolutions facilitate the formation of a hydroxide phase, namely, Ni­(OH)_2_, which is subsequently oxidized to the NiOOH phase.^13^ The online ICP-MS results confirm these mechanistic pathways, highlighting that selenium acts as a sacrificial species that drives NiOOH formation, following the proposed pathway (eq 1)­

1 $${\mathrm{NiSe}}_{2\left(s\right)}+y{\mathrm{OH}}_{\left(\mathrm{aq}\right)}^{-}\to \mathrm{Ni}{\left(\mathrm{OH}\right)}_{2\left(s\right)}+2{\mathrm{SeO}}_{x\left(\mathrm{aq}\right)}^{n-}+zH_{2}O_{\left(l\right)}$$

where *y* and *z* are the generic stoichiometric coefficients of the reaction, and SeO_*x*_^n–^ represents the selenium oxyanions, most likely as SeO_3_^2–^ under potential range before the Ni oxidation,^13^ as predicted by the Se Pourbaix diagram (Figure S9).

At this stage, the surface of the material comprises a mixture of pristine NiSe_2_ and Ni­(OH)_2_. Both species are electrochemically oxidized, resulting in the continuous formation of NiOOH by the proposed oxidation pathway (eq 2)­

2 $$x{\mathrm{NiSe}}_{2\left(s\right)}+y\mathrm{Ni}{\left(\mathrm{OH}\right)}_{2\left(s\right)}+{\mathrm{zOH}}_{\left(\mathrm{aq}\right)}^{-}\to w{\mathrm{NiOOH}}_{\left(s\right)}+a{\mathrm{SeO}}_{3\left(\mathrm{aq}\right)}^{2-}+b{\mathrm{SeO}}_{4\left(\mathrm{aq}\right)}^{2-}+cH_{2}O_{\left(l\right)}+de^{-}$$

where *x*, *y*, *z*, *w*, *a*, *b*, *c*, and *d* are the generic stoichiometric coefficients of the reaction.

This reaction suggested the progressive structural evolution of the NiSe_2_-based precatalyst, where Se leaching and Ni oxidation are strongly coupled. The result is the in situ formation of an active NiOOH phase, while selenium is leaching as SeO_3_^2–^ and SeO_4_^2–^.^13^ Once NiSe_2_ is fully oxidized, or when its NiSe_2_ becomes less accessible due to the formation of an oxidized overlayer, NiOOH formation depends on the Ni­(OH)_2_ phase, as described by the equilibrium (eq 3)­

3 $$\mathrm{Ni}{\left(\mathrm{OH}\right)}_{2\left(s\right)}+{\mathrm{OH}}_{\left(\mathrm{aq}\right)}^{-}⇌{\mathrm{NiOOH}}_{\left(s\right)}+H_{2}O_{\left(l\right)}+e^{-}$$

The thermodynamic favorability of Se leaching can be interpreted using the hard and soft acid–base (HSAB) theory. According to Marquez and Buddie,^58^ the Ni^2+^ and Ni^3+^ cations (hard acids) strongly interact with hydroxyl species (OH^–^), which are hard bases, thus forming stronger bonds than with Se_2_^2–^ (a soft base), thus favoring oxygen substitution over Se retention. This rationale is consistent with theoretical reports, according to which the substitution of Se by oxygen species is thermodynamically favorable since Ni–O bonds are stronger than Ni–Se bonds, and the adsorption of oxygenated intermediates on NiOOH active sites lowers the overall reaction free energy compared to when retaining Se^–^.^11–13^ Therefore, selenium contributes by playing a sacrificial role, promoting the structural reconstruction of NiOOH, a catalytically active phase for OER. Once dissolved, selenium no longer participates directly in the OER mechanism, but its initial presence is crucial to facilitate the formation of active sites and lower the energy barrier for catalyst activation.

By modifying the surface composition, IrO_*x*_ reduces the Ni content on the surface, consequently reducing the NiOOH available for the OER catalyst, which in turn slows Se dissolution. The reduced selenium dissolution may be attributed to the formation of a more oxidized or compact surface layer that hinders direct access to the NiSe_2_ phase, shifting the activation pathway toward the oxidation of Ni­(OH)_2_. Online ICP-MS results indicate that IrO_*x*_ dissolution occurs only during OER at potentials above ∼1.47 V_RHE_. This observation is consistent with literature reports showing that in highly alkaline media, IrO_*x*_ dissolution proceeds predominantly via the oxidation of Ir species to the Ir^6+^, forming the soluble IrO_4_^2–^ species.^39,59,60^ Our experimental findings therefore align with the expected alkaline dissolution pathway of IrO_*x*_, confirming that IrO_*x*_ remains largely stable until high overpotentials, at which point dynamic restructuring and dissolution occur. Therefore, based on the Ir Pourbaix diagram (Figure S22) and previous work,^39,60^ Ir^3+^/Ir^4+^ species in oxide form dissolve as IrO_4_^2–^, as described by eqs 4 and 5

4 $${\mathrm{Ir}}_{2}O_{3\left(s\right)}+2{\mathrm{OH}}_{\left(\mathrm{aq}\right)}^{-}\to 2{\mathrm{IrO}}_{2\left(s\right)}+H_{2}O_{\left(l\right)}+2e^{-}$$

5 $${\mathrm{IrO}}_{2\left(s\right)}+4{\mathrm{OH}}_{\left(\mathrm{aq}\right)}^{-}\to {\mathrm{IrO}}_{4\left(\mathrm{aq}\right)}^{2-}+2H_{2}O_{\left(l\right)}+2e^{-}$$

Additionally, at higher applied potentials (e.g., > 1.50 V_RHE_), Se dissolution increases again, likely due to further oxidation of bulk NiSe_2_ and generation of additional Ni^3+^ sites (eq 3). Although IrO_*x*_ itself dissolves during the OER process at high potentials, and considering the HSAB theory, Ir^4+^ species are also strong acids, while Ir^3+^ is considered an acid borderline species, exhibiting intermediate or mixed characteristics that align with the properties of hard acids.^61–63^ Therefore, these species also interact strongly and preferentially with OH^–^, and consequently, their gradual leaching reflects competitive surface reactions during OER. As illustrated in Figure 6a, the proposed surface reconstruction reaction involves the oxidation of NiSe_2_ (light green lines), OER (green lines), and the dissolution (pink lines), demonstrating how the structural reconstruction can proceed concurrently with catalytic operation.

**Figure 6:**
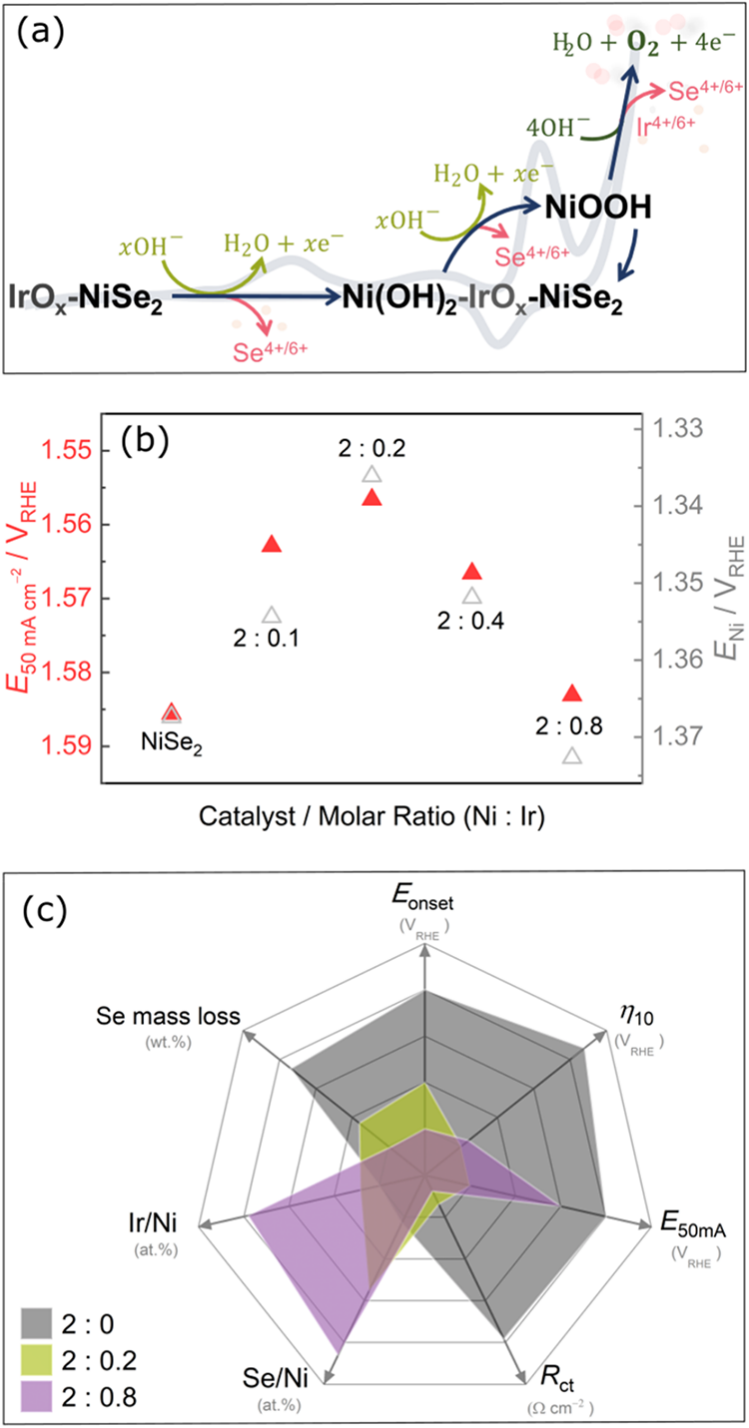
(a) Schematic representation of structural reconstruction and degradation pathways of precatalyst during the OER. (b) Relationship between *E*_Ni_ and catalytic activity of catalysts depending on the Ni:Ir ratio in the IrO_*x*_-NiSe_2_. (c) Radar chart of pristine NiSe_2_ and IrO_*x*_-NiSe_2_ with Ni:Ir ratios of 2:0.2 and 2:0.8, in terms of the values of *E*_onset_ (V_RHE_), η_10_ (V_RHE_), *E*_50 mA_ (V_RHE_), *R*_ct_ (Ω cm^2^), Se/Ni (at. %), Ir/Ni (at. %), and Se mass loss (wt %).

IrO_*x*_ incorporation enhanced the electrocatalytic activity for the OER, particularly at moderate IrO_*x*_ loadings. Two primary effects appear to contribute to this improvement. First, changes in the chemical composition: increasing IrO_*x*_ content reduces particle size, which increases the ECSA during structural reconstruction. However, excessive IrO_*x*_ coverage at higher loadings decreases the amount of Ni present on the surface, leading to the formation of segregated IrO_*x*_ phases, as suggested by electrochemical analysis (*Q*_Ni_) and XPS, respectively. Second, the incorporation of IrO_*x*_ modifies the local electronic environment and adsorption properties, reflected by the lower OER *E*_onset_ and by shifts in the Ni^2+^/Ni^3+^ redox potential. These effects are consistent with previous reports,^17,45,64,65^ which indicate that iridium and iridium oxides incorporate to nickel-based catalyst tunes the adsorption energy of oxygen intermediates. Even a low amount of IrO_*x*_ results in change in the catalytic performance of NiSe_2_. However, these beneficial effects do not follow a linear correlation.

This nonlinear behavior is further reflected in the evolution of OER performance (represented by *E*_50_), which exhibits a volcano-type trend as the IrO_*x*_ proportion increases (Figure 6b), indicating that the sample with a 2:0.2 Ni:Ir molar ratios (corresponding to 2.4 at. % Ir in IrO_*x*_-NiSe_2_) achieves a balance between chemical composition modification and activity enhancement. Similar volcano-type behavior was reported for Ir–Ni systems,^17^ where an optimal Ir fraction promotes synergistic adsorption of OH^–^ and O^–^ intermediates at the IrO_*x*_/NiOOH interface, and at the same time, higher IrO_*x*_ loadings lead to segregated IrO_*x*_ phases that compromise interfacial effects and reduce activity. Further studies are needed to determine whether a direct correlation exists between the Ni oxidation potential and OER activity.

Overall, the trend highlights trade-off between the beneficial and detrimental effects of IrO_*x*_ incorporation. Moderate IrO_*x*_ loadings promote (i) an increase in surface area, (ii) maintenance of the oxidation of Ni into active oxyhydroxides phase, and (iii) optimized adsorption and coupling of OER intermediates at the interface. However, excessive IrO_*x*_ incorporation leads to (iv) a reduction in the number of accessible NiOOH active sites, as evidenced by the progressive increase in the Ir/Ni atomic ratio (Table S7) and by the corresponding decrease in Ni atomic percentage at the surface, despite bulk Ni content remaining constant (Table S1). This trend supports the occurrence of a site-blocking effect, consistent with the volcano-shaped dependence of activity on IrO_*x*_ loading. Therefore, the optimal catalytic performance arises from a balance between maximizing synergistic interactions and preserving the accessibility to NiOOH active centers within the catalyst. The results underscore the importance of controlling the chemical and structural composition to balance the interaction between the catalyst and reaction intermediates, as predicted by the Sabatier principle,^66^ which maximizes catalytic efficiency when the interaction between the catalyst and reaction intermediates is balanced.

The variation of the LSV current densities further corroborates this behavior. Although IrO_*x*_ addition lowers the *E*_onset_, higher IrO_*x*_ loadings lead to reduced anodic current density at elevated overpotentials (e.g., *E*_50_), suggesting kinetic limitations. These could originate from hindered oxygen desorption and less efficient electron transfer pathways.^48^ Such an effect is consistent with the observed enrichment of IrO_*x*_ at the surface, which strengthens OH^–^ adsorption but simultaneously decreases the exposure of NiOOH active sites. In summary, the results suggest that activity may be influenced by the strength of interaction with OER intermediates. Moderate IrO_*x*_ loadings optimize this interaction, improving adsorption and lowering *E*_onset_, whereas excessive IrO_*x*_ strengthens OH^–^ adsorption, hindering desorption process. This trade-off is consistent with the Sabatier principle,^66^ as also reflected in the volcano-type dependence of OER performance on IrO_*x*_ content (Figure 6b).

A radar chart was constructed to have a better overview of the IrO_*x*_ loading effect on the different parameters evaluated for the OER, a (Figure 6c). Note that, to complement the data set used for the radar chart, an additional online ICP-MS dissolution measurement was performed for the IrO_*x*_-NiSe_2_ (Ni:Ir ratio of 2:0.8) catalyst using the *Low scan rate protocol* to obtain the Se mass loss. The corresponding results are shown in Figure S23. Compared to the pristine NiSe_2_, one notices that IrO_*x*_-NiSe_2_ with Ni:Ir ratio of 2:0.2, resulted in lowering the *E*_onset_, η_10_, *E*_50 mA_, and *R*_ct_ (Figure 6c). This improved OER performance is associated with two factors, the first one is the structural reconstruction of the IrO_*x*_-NiSe_2_ to form the IrO_*x*_-NiOOH due to oxidation and leaching of selenium species from the precatalyst. The second factor that favored improvement of the OER is the suitable superficial chemical composition of the catalyst, namely, an optimized amount of NiOOH and IrO_*x*_ active sites that can synergistically enhance the OER catalysis. For the IrO_*x*_-NiSe_2_ with the highest IrO_*x*_ loading (2:0.8), despite the decrease in *E*_onset_, *R*_ct_, and selenium occurrence of structural reconstruction, compared to that of the pristine NiSe_2_, the *E*_50 mA_ considerably increased, and no significant variation was observed for the η_10_ in relation to the sample with Ni:Ir ratio of 2:0.2. The decrease in electrocatalytic activity for the OER was assigned to the superficial accumulation of IrO_*x*_ over the NiOOH active sites (i.e., site-blocking effect), as evidenced by the increase in Ir/Ni and Se/Ni atomic ratios from XPS analyses (Figure 6c), which decrease the accessibility of the sites on the surface, and also affect the adsorption process of OER intermediates and O_2_ desorption (as discussed in the electrochemical analyses).

Regarding the AST CV-based experiments conducted using RDE and SFC-ICP-MS, these analyses confirmed the resistance of the NiOOH active phase under harsh electrolysis conditions. However, the precatalyst undergoes complete oxidation and additional structural transformation, including particle coalescence and a likely reduction in surface area. Notably, IrO_*x*_-modified materials exhibit attenuated Se leaching and slower degradation, suggesting that IrO_*x*_ incorporation contributes to improved structural robustness under prolonged operation. Lastly, a comparative analysis of the OER activity and stability of the IrO_*x*_-NiSe_2_ catalysts was carried out with the reported catalysts in the literature. For this analysis, it is worth mentioning that it is difficult to make a precise comparison because of the different electrochemical experimental conditions and surface characteristics of the reported catalysts. As shown in Table S10, the IrO_*x*_-NiSe_2_ (Ni:Ir ratio of 2:0.2) catalyst exhibited improved stability compared to pristine NiSe_2_, evidencing the mitigating structural degradation due to IrO_*x*_ incorporation. However, when compared with the most stable NiFeCo-based materials systems,^67^ the durability of IrO_*x*_-NiSe_2_ is still limited. In addition, differences may arise from the substrate effect. For instance, NiSe_2_ catalysts supported on conductive substrates such as Au^68^ or Ni foam^69^ have demonstrated enhanced long-term stability. These findings suggest that the stability of IrO_*x*_-NiSe_2_ could be further optimized through substrate engineering or interfacial modifications, aspects that are beyond the scope of the present work. Regarding the comparative OER activity analysis, the η_10 mA_ for the OER of the IrO_*x*_-NiSe_2_ with Ni:Ir ratio of 2:0.2 (η_10 mA_ = 285 mV) is very close to the reported value for the benchmark NiFe-based oxides and hydroxides (η_10 mA_ ∼ 280 mV), which is recognized as one of the most active OER catalysts in alkaline media.^70^ This comparison highlights that nickel-based chalcogenides, although less explored than NiFe oxides, can achieve similar performance, reinforcing their potential as relevant precatalysts for OER. In this context, future studies involving chalcogenide-based FeNi materials are particularly promising, as they could combine the high intrinsic activity of NiFe systems with the favorable reconstruction and stability features provided by chalcogenide-based frameworks.

## 4 Conclusion

This work presents the synthesis, structural characterization, and electrochemical evaluation of NiSe_2_ precatalysts modified with IrO_*x*_ nanoparticles for the alkaline OER. The incorporation of small amounts of IrO_*x*_ influenced particle nucleation, resulting in reduced particle size, increased surface area, and improved catalytic activity. These modifications not only facilitate the adsorption of OER intermediates but also reduce the amount of Ni species available on the surface, which consequently harms catalytic activity. Online dissolution analysis confirmed that selenium acts as a sacrificial species to form the active NiOOH phase, which is rationalized by HSAB theory, wherein the substitution of Se_2_^2–^ by oxygen species is driven by the favorable interaction with Ni^2+^, promoting the formation of the NiOOH active phase. The Ni:Ir ratio was found to influence the OER performance. At an optimal composition (IrO_*x*_-NiSe_2_ with 2.4 at. % Ir), IrO_*x*_ incorporation improves the adsorption of reaction intermediates without blocking active sites. In contrast, higher IrO_*x*_ contents promote excessively strong adsorption, reducing OER efficiency, which is consistent with Sabatier’s principle. The results highlight a compositional strategy to optimize active surface areas and intermediate adsorption properties in nickel chalcogenides. Moderate IrO_*x*_ incorporation enhances catalytic activity while preserving surface stability. Our work not only guides the rational design of OER precatalysts for alkaline electrolysis but also provides the oxidative reconstruction pathways in transition metal chalcogenides, particularly of nickel selenides by dissolution under anodic conditions.

## 5 Experimental Section

### 5.1 Synthesis of NiSe_2_

The NiSe_2_ supported on Black Pearls 2000 carbon (CB) was prepared by the hydrothermal method. A dispersion of 35 mg carbon and 2 mmol of Ni­(C_2_H_4_O_2_)_2_ was prepared in 20 mL ultrapure water (18 MΩ cm^–2^). In another flask, 4 mmol of Se powder and 6 mmol of NaBH_4_ were added in 20 mL ultrapure water; after 1 h of vigorous stirring, the solution turned to a dark wine color. The flask containing Ni/CB was then added to the reduced selenium, and the volume was adjusted with ultrapure water to a final quantity of 60 mL. After 1 h of stirring, the solution was transferred to a Teflon bottle and hydrothermally treated at 180 °C for 7 h. A black precipitate was obtained, which was washed by centrifugation with ethanol and ultrapure water, filtered with boiling water, and dried at 80 °C for 2 h.

### 5.2 Synthesis of IrO_*x*_-NiSe_2_

For the synthesis process of IrO_*x*_-modified NiSe_2_, different proportions of suspended iridium oxide nanoparticles were added before the hydrothermal step described above. The iridium oxide nanoparticles were prepared according to the method reported by da Silva et al.^20^ Briefly, it was taken from the H_2_IrCl_6_ stock solution (concentration of 50 mg mL^–1^) different aliquots (i.e., 0.5, 1.0, 8.0, and 30 μL), and each were subjected to alkaline hydrolysis by slowly adding a 1 mol L^–1^ LiOH solution (i.e., 0.6, 1.2, 2.4, and 4.8 mL, respectively). The LiOH solution was added to the Ir^4+^ solutions using a syringe pump at a rate of 0.2 mL h^–1^ to form suspensions containing IrO_*x*_ nanoparticles. After the hydrolysis process, each of the IrO_*x*_ suspensions were combined with 40 mL of a solution containing 2 mmol of Ni­(C_2_H_4_O_2_)_2_, 35 mg of CB, 4 mmol of Se powder, and 6 mmol of NaBH_4_ (as described in Synthesis of NiSe_2_). Sequentially, each of these mixtures was diluted to a final volume of 60 mL with ultrapure water to achieve different amount of IrO_*x*_ (i.e., 0.1, 0.2, 0.4, and 0.8 mmol of Ir^4+^). Lastly, these mixtures were stirred for 1 h, and then subjected to the hydrothermal step (180 °C for 7 h). Other synthesis details can be found in Section S1 of the Supporting Information.

### 5.3 Material Characterization

The materials were characterized by X-ray powder diffractometry (XRD) on D8 Advance (Bruker) equipment using Cu *Kα* radiation (1.5418 Å) in the range of 20°–70° at a scan rate of 0.075° s^–1^. The diffraction peaks were qualitatively identified using the database reference standards, the Joint Powder Diffraction Patterns Committee (JCPDS). The average crystallite sizes (*d*) in nanometers were calculated for each sample using the Scherrer equation^22^ (eq 6) based on the XRD patterns

6 $$d=\frac{K\lambda }{\beta \cos\theta }$$

where *K* is the shape factor (taken as 0.9), λ is the wavelength of the Cu *Kα* radiation, θ is the Bragg diffraction angle, and β is the full width at half-maximum (fwhm) of the diffraction peak. The Scherrer equation was applied to the most intense, well-defined, and symmetric peak (around 33.7°).

The elemental composition of the catalysts was determined using an energy-dispersive X-ray spectrometer, model XFLASH 6–60 (Bruker), on a field emission scanning electron microscope (FEG-SEM) JEOL JMS 7200 equipment. The morphology was analyzed by a transmission electron microscope (TEM) JEOL JEM-210 with a LaB_6_ filament and a voltage of 200 kV. The samples were prepared by dispersing the catalysts in isopropanol and depositing a drop of this dispersion on a copper grid (EMS, 400 mesh). The composition was analyzed by scanning transmission electron microscopy (STEM) using EDS mapping coupled to TEM. The surface composition and the electronic structure were analyzed by X-ray photoelectron spectrometer (XPS), using a ThermoScientific K-α+ spectrometer with an aluminum monochromator. All obtained XPS spectra were calibrated considering the energy of the C 1s peak to be 284.5 eV.

### 5.4 Electrochemical Measurements

Electrochemical measurements were conducted in a three-electrode configuration employing a rotating disc electrode (RDE) and an AUTOLAB potentiostat. A graphite rod was used as a counter electrode, and an Hg/HgO electrode was used as a reference electrode. The Hg/HgO reference electrode potential was previously measured vs RHE, resulting in *E*_Hg/HgO_ vs *E*_RHE_ = 0.900 V (in 1 mol L^–1^ KOH), and all potential values recorded *vs E*_Hg/HgO_ electrode were subsequently recalibrated to the scale of RHE. The catalyst ink was prepared by dispersing 2 mg of catalyst in 1 mL of isopropanol and 10 μL of Nafion solution (5 wt %, Alfa Aesar). A 15 μL aliquot of the suspension was drop-cast onto a glassy carbon disk (5 mm diameter, geometric area of 0.196 cm^2^). To induce the electrochemical reconstruction of the catalyst, ten cyclic voltammetry (CV) scans were first conducted from 0.9 to 1.6 V_RHE_ at a scan rate of 100 mV s^–1^. After this activation step, linear sweep voltammetry (LSV) was performed from 0.9 to 1.7 V_RHE_ at a scan rate of 5 mV s^–1^ to assess the OER activity. The ohmic resistance (*R*_Ω_) was determined by electrochemical impedance spectroscopy (EIS) at open circuit potential in the frequency range of 100 kHz to 0.1 Hz, and amplitude of 10 mV. All LSV curves were corrected for 90% of the *iR* drop. EIS was also carried out at 1.47 V_RHE_ to access the interfacial charge transfer properties under applied potential. The electrochemically active surface area (ECSA) was estimated from the double-layer capacitance (*C*_d_) obtained by CV in a nonfaradaic potential range from 0.629 to 0.682 V_RHE_ (−0.271 to – 0.218 *V*_Hg/HgO_) at different scan rates (100, 125, 150, 200, and 300 mV s^–1^). The anodic current density (*j*_a_) and cathodic current density (*j*_c_) were taken from the middle of the potential range of the CVs (i.e., 0.66 V_RHE_) and then plotted as (*j*_a_ – *j*_c_)/2 vs. sweep rate (υ). The estimated *C*_d_ value corresponded to the slope of the linear equation of the (*j*_a_ – *j*_c_)/2 vs υ plots.^50^ Additionally, the stability of the catalysts was evaluated following an AST protocol, which consisted of 500 CV scans performed at a scan rate of 100 mV s^–1^, within a potential window of 0.3 to 1.5 V_RHE_. Stability experiments were also performed via polarizing galvanostatically the catalysts at 10 mA cm^–2^ over 10 h of experiment. Before running this stability experiment, the catalysts were submitted to 10 CVs which aimed to perform the structural reconstruction. These measurements were carried out with a RDE in Ar-saturated 1 mol L^–1^ KOH electrolyte.

### 5.5 Online Analysis Dissolution by Scanning Flow Cell Coupled with an ICP-MS

Online dissolution measurements were performed using a SFC coupled to an ICP-MS (PerkinElmer NexION 350). The SFC was configured with a graphite rod as the counter electrode and an Ag/AgCl (3 mol L^–1^, Metrohm) electrode as the reference (*E*_RHE_ = 0.960 V in 0.1 mol L^–1^ KOH). The working electrode was prepared by drop-casting 0.25 mL of catalyst ink (1.3 mg mL^–1^, 12% isopropanol, 87% ultrapure water, and 0.3% Nafion solution 5 wt %) on a glassy carbon (GC) substrate (5 × 5 cm^2^, Sigradur G, HTW), previously polished. The ink was formulated at a concentration of 1.3 mg mL^–1^ using a solvent mixture composed of approximately 12% isopropanol, 87% ultrapure water, and 0.3% Nafion solution (5 wt %) by volume. The pH of the ink suspension was adjusted to ∼10 using 1 M KOH, prior to deposition. The target catalyst loading was 20–25 μg cm^–2^, and the geometric area of each spot (average 0.0133 ± 0.0021 cm^2^) was measured using a laser confocal microscope (Keyence VK-X250). All electrochemical and dissolution data were normalized by the geometric surface area. The samples were placed on an XYZ positioning stage (Physik Instrumente M-403), and electrical contact was established using a metallic probe pressed onto the surface of the GC as the working electrode. Details of the SFC-ICP-MS setup and operation are described in more detail in a previously published work.^19^

The ICP-MS was operated in dynamic reaction cell (DRC) mode with methane (CH_4_, 4.5 purity, Air Liquide) to suppress polyatomic interferences such as ^40^Ar^16^O^+^, an internal standard (IS) solution containing ^74^Ge and ^187^Re (50 μg L^–1^) was prepared in 2% HNO_3_ and 5% methanol. The ICP-MS was calibrated daily using a four-point calibration curve for ^60^Ni, ^77^Se, and ^207^Ir, with a concentration range of 0–5 μg L^–1^ in 0.1 mol L^–1^ KOH. The ^207^Ir was quantified by reaction with methanol from the IS. The electrolyte in the SFC was mixed with the IS via a Y-connector before entering the ICP-MS. All measurements were conducted in Ar-saturated 0.1 mol L^–1^ KOH.

To investigate the dissolution behavior during structural reconstruction and OER processes, a series of electrochemical protocols were developed:

(a) *Low scan rate protocol*: A single CV scan from 0.3 to 1.5 V_RHE_ at 2 mV s^–1^ was employed to resolve the dissolution events of Ni, Se, and Ir associated with redox transitions. This low scan rate minimizes capacitive current contributions and enables the distinction of closely spaced dissolution peaks.
(b) *Fast scan rate protocols*: Designed to assess dissolution under accelerated cycling conditions using 10 consecutive CV cycles at 100 mV s^–1^, with increasing potential windows: *Protocol I*, from 0.3 to 1.2 V_RHE_; *Protocol II*, from 0.3 to 1.4 V_RHE_; and *Protocol III* from 0.3 to 1.6 V_RHE_.
(c) *Post-Protocol III*: A LSV scan from 0.3 to 1.7 V_RHE_ at 5 mV s^–1^ was conducted after *Protocol III*.
(d) *AST protocol*: A total of 500 CV scans from 0.3 to 1.5 V_RHE_. A total of 500 CV cycles from 0.3 to 1.5 V_RHE_ at 100 mV s^–1^ was applied to simulate long-term electrochemical degradation.

All protocols were initiated and finalized with a 5 min hold at 0.3 V_RHE_. The mass loss of Ni, Se, and Ir was estimated by integrating the dissolution profiles and normalizing using the wt % of each element determined by EDS analysis.

### 5.6 Post-AST Structural and Compositional Analysis

To evaluate morphological and compositional changes induced by electrochemical degradation under long-term oxidation conditions, EDS and identical location transmission electron microscopy (IL-TEM) analyses were performed before and after the AST protocol in 1 mol L^–1^ KOH. EDS analysis was carried out with the catalyst directly drop-cast onto the GC substrate of the RDE working electrode before and after the AST protocol, as described in the Section 5.4. IL-TEM was conducted using the same transmission electron microscope described in the Section 5.3. For this purpose, the catalyst dispersion was prepared and drop-casting deposited onto carbon-coated gold TEM grids (EMS, 300 mesh). Morphological changes and the formation of potential oxidation sites were evaluated by imaging the exact location on the grid before and after the AST.

## Supplementary Material



## Notes

Scheme of hydrothermal synthesis; EDS, TEM and XPS results; Electrochemical measurements results; Pourbaix diagram of Ni, Se, and Ir; Total dissolution of Ni, Se, and Ir under varied voltammetric protocols; Dissolution profiles of NiSe_2_ and IrO_*x*_-NiSe_2_ under voltametric and AST protocols; Determination of onset OER potential (PDF)
