# Supplementary material for: Compositional Engineering of NiSe2 Precatalysts with IrO x for Controlled Reconstruction toward Improved Alkaline OER
Source: ACS Appl Mater Interfaces. 2025 Nov 23;17(49):66554–68. doi: 10.1021/acsami.5c15821 (PMC12874214; doi:10.1021/acsami.5c15821)
Supplement: Supplementary file 1 [file am5c15821_si_001.pdf]

Supporting Information

Compositional Engineering of $NiSe_2$ Precatalysts with IrO_x for Controlled Reconstruction towards Improved Alkaline OER

Pâmella S. Rodrigues[1,2], Tatiana Priamushko[2], Moisés A. de Araújo[1], Gabriel C. da Silva[3], Serhiy Cherevko[2], Edson A. Ticianelli[1]**

[1]São Carlos Institute of Chemistry, University of São Paulo, Av. Trab. Sancarlense, 400, 13566-590, São Carlos, Brazil.

[2]Helmholtz-Institute Erlangen-Nürnberg for Renewable Energy (IET-2), Forschungszentrum Jülich GmbH, Cauerstr. 1, 91058 Erlangen, Germany

[3]Chemistry Department, Federal University of Viçosa, Av. Peter Henry Rolfs, 36570-900, Viçosa, Brazil.

*Corresponding Autor: Edson A. Ticianelli (edsont@iqsc.usp.br) and Serhiy Cherevko (s.cherevko@fz-juelich.de)

Contents

S1. Hydrothermal synthesis of nickel selenide 5
S2. Structure Characterization 6
Table S1. Chemical composition in terms of weigh percentage (wt.%) and atomic percentage (at.%) from the EDS analysis of pristine $NiSe_2$ and IrO_x-$NiSe_2$ with different molar ratio of Ni:Ir obtained by field emission scanning electron microscope (FEG-SEM). 6

Figure S2. EDS elemental mapping of (a) pristine $NiSe_2$ and (b) IrO_x-$NiSe_2$ synthesized with a Ni:Ir molar ratio of 2:0.2 obtained by transmission electron microscope (TEM). 6

Figure S3. TEM micrograph of IrO_x-$NiSe_2$ with molar ratio of 2:0.8 (Ni:Ir). 7

Figure S4. High-resolution XPS spectra for (a-c) Ni 2p, (d-f)Se 3d, (e-f) Ir 4f, (g-i) C 1s and (j-l) O1s core levels of pristine $NiSe_2$ and IrO_x-$NiSe_2$ with Ni:Ir molar ratios of (a, d, g, j) 2:0 ($NiSe_2$ pristine), (b, e, h, k) 2:0.2 and (c, f, i, l) 2:0.8. 8

Figure S5. High-resolution XPS spectra of (a) Ir 4f and (b) O 1s core levels of pristine IrO_x. 9

Table S2. Fit parameters employed for modeling peaks and baseline of the XPS spectra of pristine $NiSe_2$. 9

Table S3. Fit parameters employed for modeling peaks and baseline of the XPS spectra of pristine IrO_x. 10

Table S4. Fit parameters employed for modeling peaks and baseline of the XPS spectra of IrO_x-$NiSe_2$ (Ni:Ir ratio of 2:0.2). 10

Table S5. Fit parameters employed for modeling peaks and baseline of the XPS spectra of IrO_x-$NiSe_2$ (Ni:Ir ratio of 2:0.8). 11

Table S6. XPS peaks assignment of pristine $NiSe_2$ and IrO_x, and IrO_x-$NiSe_2$ with Ni:Ir molar ratios of 2:0.2 and 2:0.8. 12

Table S7. Atomic percentage from XPS analyses of pristine $NiSe_2$ and IrO_x-NiSe with Ni:Ir molar ratios of 2:0.2 and 2:0.4. 12

S3. Structural reconstruction and OER performance 13
Figure S6. Structural reconstruction via cyclic voltammograms at 100 mV s^{-1} of IrO_x-$NiSe_2$ with different molar ratios of Ni:Ir with a highlight of the oxidation peaks of Se (▲) and Ni (♦). The electrolyte was an Ar-saturated solution of 1 mol L^{-1} KOH. 13

Figure S7. (a) Variation of electrochemically active Ni species during structural reconstruction via ten scans cycles at 100 mV s^{-1} of $NiSe_2$ and IrO_x-$NiSe_2$ with 2:0.2 and 2:0.8 molar ratios of Ni:Ir.* (b) Cathodic and anodic integrate charge density of nickel reduction and oxidation at tenth scan cycle for all samples 14

Table S8. Summary of electrochemical parameters for $NiSe_2$ and IrO_x-$NiSe_2$ with different molar ratios of Ni:Ir catalysts in 1 mol L^{-1} KOH 15

Figure S8. Nickel Pourbaix diagrams calculated from experimental thermodynamic tables[11] with aqueous ion concentrations 10^{-5} mol L^{-1} at 25 °C. The pink area highlighted indicates the potential range performed in this work (0.3 - 1.6 V_{RHE}, E_{SHE}=E_{RHE} – 0.059pH), considering a pH between 13 and 14. The light dashed line (*1'*) indicates the boundaries of the regions where the dissolved substances are relatively predominant (*1':* Ni^{2+} / $HNiO^{-}_2$, pH=10.13) 16

Figure S9. Selenium Pourbaix diagrams calculated from experimental thermodynamic tables[11] with aqueous ion concentrations 10^{-5} mol L^{-1} at 25 °C. The pink area highlighted

indicates the potential range performed in this work (0.3 - 1.6 V_{RHE}, E_{SHE}=E_{RHE} – 0.059pH), considering a pH region between 13 and 14. 17

Figure S10. Total dissolution of Se and Ir during the CV protocol from 0.3–1.5 V_{RHE} at lower scan rate (2 mV s^{-1}). The white bars represent the total dissolution normalized by the weight percent (wt.%) of Se and Ir obtained by EDS analysis. 18

Figure S11. Dissolution of (b) Ni, (c) Se, and (d) Ir during (a) *Protocol I* (0.3–1.2 V_{RHE}) at 100 mV s^{-1}. The electrolyte was an Ar-saturated solution of 0.1 mol L^{-1} KOH. 18

Figure S12. Total dissolution of Se and Ir during *Protocol I* (0.3–1.2 V_{RHE}), *Protocol II* (0.3–1.4 V_{RHE}) and Protocol III (0.3–1.6 V_{RHE}). The white bars represent the total dissolution normalized by the weight percent (wt.%) of Se and Ir obtained by EDS analysis. 19

Figure S13. Determination of E_{onset} using the tangent method (current density window from 0 to 10 mA cm^{-2}) from the LSV at 5 mV s^{-1}. This method was chosen due to the non-negligible current density observed between the Ni oxidation peak (~1.35 V, Figure S6) and the onset of the OER. This residual current density is likely associated with pseudocapacitive behavior, and/or early adsorption of OER intermediates. Additionally, increased capacitive contributions may arise from the high surface area and retention of OER intermediates after surface reconstruction. 20

Figure S14. Dissolution of Ni, Se, and Ir during the LSV at 5 mV s^{-1} following the reconstruction process (*Protocol III*) in Ar-saturated 0.1 mol L^{-1} KOH solution. 21

Figure S15. Complex-plane impedance and Bode diagrams at 1.47 V_{RHE} obtained after 10 CVs for (a-b) $NiSe_2$, IrO_x and (d-e) IrO_x-$NiSe_2$ with different molar ratios of Ni:Ir. The equivalent circuit used for fitting the impedance of spectra for (c) $NiSe_2$ and IrO_x, and (f) IrO_x-$NiSe_2$ with different molar ratio of Ni:Ir. The electrolyte was an Ar-saturated solution of 1 mol L^{-1} KOH. 22

Figure S16. (a-e) Cyclic voltammograms obtained at different υ (100, 125, 150, 200, and 300 mV s^{-1}) and in a non-faradaic potential range (from 0.629 to 0.682 V_{RHE}) of $NiSe_2$ and IrO_x-$NiSe_2$ with different molar ratios of Ni:Ir. This analysis was performed without the 10 CVs (i.e., the reconstruction process). (f) (j_a – j_c)/2 vs. υ plots of $NiSe_2$ and IrO_x-$NiSe_2$ with different molar ratio of Ni:Ir. The electrolyte was an Ar-saturated solution of 1 mol L^{-1} KOH. 24

Figure S17. TOF values at different applied potentials of pristine $NiSe_2$ (Ni:Ir ratio of 2:0) and IrO_x-$NiSe_2$ with different molar ratios of Ni:Ir. The electrolyte was an Ar-saturated solution of 1 mol L^{-1} KOH. 25

Figure S18. Accelerated stress test using a rotating disc electrode by cyclic voltammograms at 100 mV s^{-1} for (a) pristine $NiSe_2$ and (c) IrO_x-$NiSe_2$ (Ni:Ir ratio of 2:0.2) and their respective (b, d) linear sweep voltammograms at 1 mV s^{-1}. The electrolyte was an Ar-saturated solution of 1 mol L^{-1} KOH. 26

Figure S19. Dissolution of (a) Ni, (b) Se, and (c) Ir during AST performed using SFC-ICP-MS in Ar-saturated solution of 0.1 mol L^{-1} KOH. 26

Figure S20. Total dissolution of Se and Ir during AST protocol. The white bars represent the total dissolution normalized by the weight percent (wt.%) of Se and Ir obtained by EDS analysis. 27

Figure S21. Chronopotentiometry curves at 10 mA cm^{-2} of pristine $NiSe_2$ and IrO_x-$NiSe_2$ (Ni:Ir ratio of 2:0.2) in a RDE setup. The electrolyte was an Ar-saturated solution of 1 mol L^{-1} KOH. 27

Figure S22. Iridium Pourbaix diagrams calculated from experimental thermodynamic tables[11] with aqueous ion concentrations 10^{-5} mol L^{-1} at 25 °C. The pink area highlighted indicates the potential range performed in this work (0.3 - 1.6 V_{RHE}, E_{SHE}=E_{RHE} – 0.059pH), considering a pH region between 13 and 14. The light dashed line (*1'*) indicates the boundaries of the regions where the dissolved substances are relatively predominant (*1'*: Ir^{3+} / IrO_4^{2-}, $E^{\ominus}$=1.448 – 0.1576pH)..28

Table S10. Comparison of OER performance of nickel-based catalyst in 1 mol L^{-1} KOH alkaline conditions. 29

Figure S23. Dissolution of Ni, Se, and Ir for IrO_x-$NiSe_2$ with 2:0.8 ratio of Ni:Ir during the protocol at lower scan rate (2 mV s^{-1}) performed using SFC-ICP-MS in Ar-saturated solution of 0.1 mol L^{-1} KOH. 30

S1. Hydrothermal synthesis of nickel selenide

The synthesized IrO_x-$NiSe_2$ materials were obtained with different molar ratio of Ni:Ir in the composition of the catalysts. The initial stages of the synthesis to obtain pure $NiSe_2$ involved the reduction of $Se_{(s)}$ by $NaBH_4$, and reacting with the Ni precursor in solution, the formation of nickel selenide occurs through the proposed mechanism as shown in Equation S1[1]:

$$Ni^{2+}_{(aq)} + 2Se_{(s)} + 2BH^{-}_{4(aq)} + 6H_2O_{(l)} \longrightarrow NiSe_{2(s)} + 2H_3BO_{3(aq)} + 7H_{2(g)} \qquad (S1)$$

The iridium oxide nanoparticles were obtained from the alkaline hydrolysis of the Ir^{4+} precursor according to the reaction in Equation S2[2,3]:

$$[IrCl_6]^{2-}_{(aq)} + yOH^{-}_{(aq)} \longrightarrow IrO_{x(s)} + 6Cl^{-}_{(aq)} + zH_2O_{(l)} \qquad (S2)$$

where *y* and *z* are the generic stoichiometric coefficients of the reaction.

According to the procedure described in the experimental section, after stirring the $NiSe_2$ suspension for 1 h, the IrO_x nanoparticle dispersion was added and stirred again. The combined mixture was then subjected to hydrothermal treatment at 180 °C for 7 h. The resulting black precipitation was washed and dried. **Figure S1** shows a schematic representation of synthesis procedures.

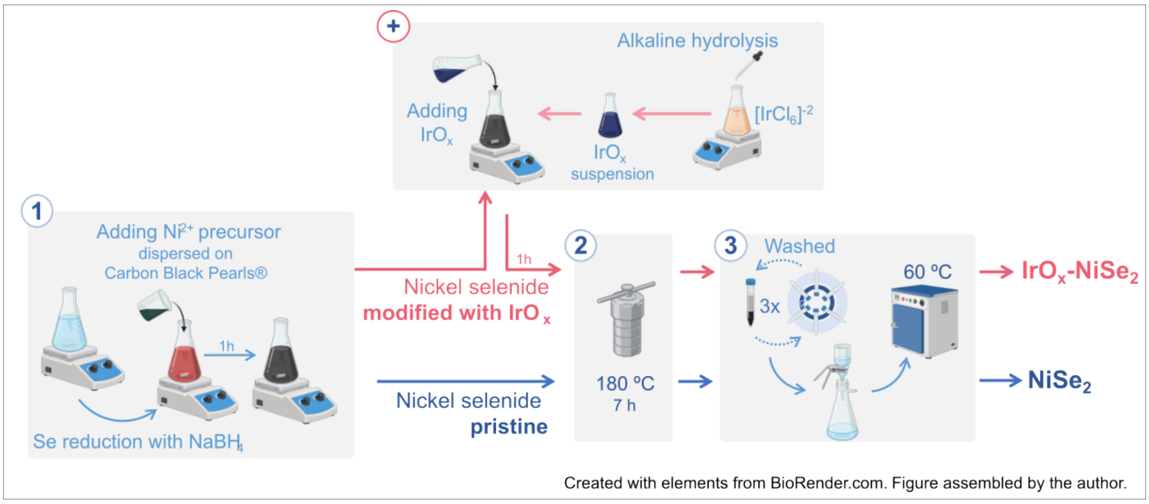

Figure S1. Procedure for the hydrothermal synthesis of $NiSe_2$ modified with nanoparticles of IrO_x nanoparticles.

S2. Structure Characterization

Table S1. Chemical composition in terms of weigh percentage (wt.%) and atomic percentage (at.%) from the EDS analysis of pristine $NiSe_2$ and IrO_x-$NiSe_2$ with different molar ratio of Ni:Ir obtained by field emission scanning electron microscope (FEG-SEM).

Catalysts (Ni:Ir)	wt. %			at. %		
	Ni	Se	Ir	Ni	Se	Ir
2 : 0 (pristine $NiSe_2$)	28.1 ± 0.56	71.9 ± 0.67	0	34.2 ± 0.54	65.8 ± 0.86	0
2 : 0.1	31.6 ± 0.42	65.1 ± 0.60	3.3 ± 0.07	39.2 ± 0.27	59.6 ± 0.67	1.2 ± 0.02
2 : 0.2	31.5 ± 1.58	62.1 ± 0.94	6.4 ± 0.05	39.3 ± 1.71	58.3 ± 0.26	2.4 ± 0.02
2 : 0.4	28.1 ± 0.93	57.4 ± 0.74	14.5 ± 0.18	37.4 ± 0.74	56.7 ± 0.55	5.9 ± 0.06
2 : 0.8	24.3 ± 1.15	54.3 ± 0.49	21.4 ± 0.04	34.1 ± 1.28	56.7 ± 0.13	9.2 ± 0.08

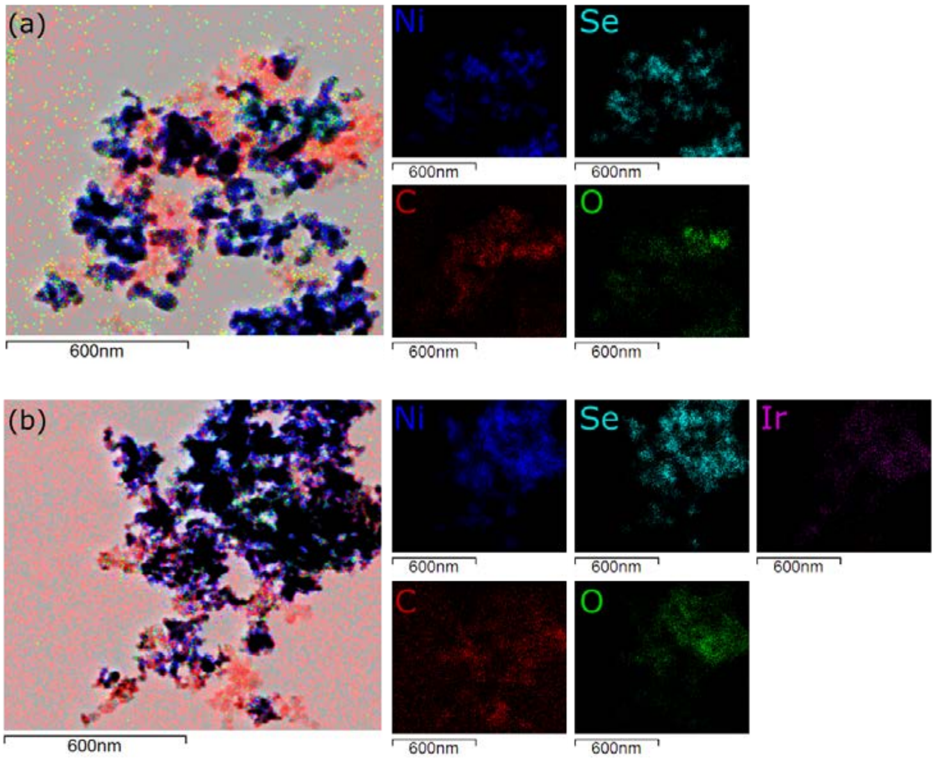

Figure S2. EDS elemental mapping of (a) pristine $NiSe_2$ and (b) IrO_x-$NiSe_2$ synthesized with a Ni:Ir molar ratio of 2:0.2 obtained by transmission electron microscope (TEM).

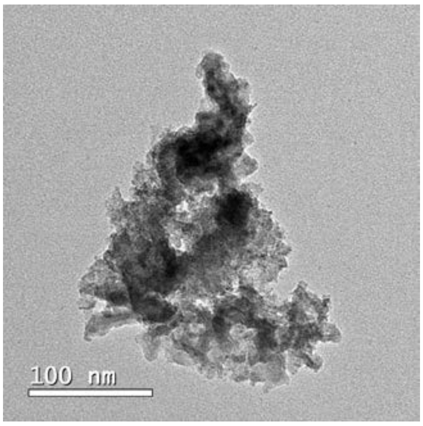

Figure S3. TEM micrograph of IrO_x-$NiSe_2$ with molar ratio of 2:0.8 (Ni:Ir).

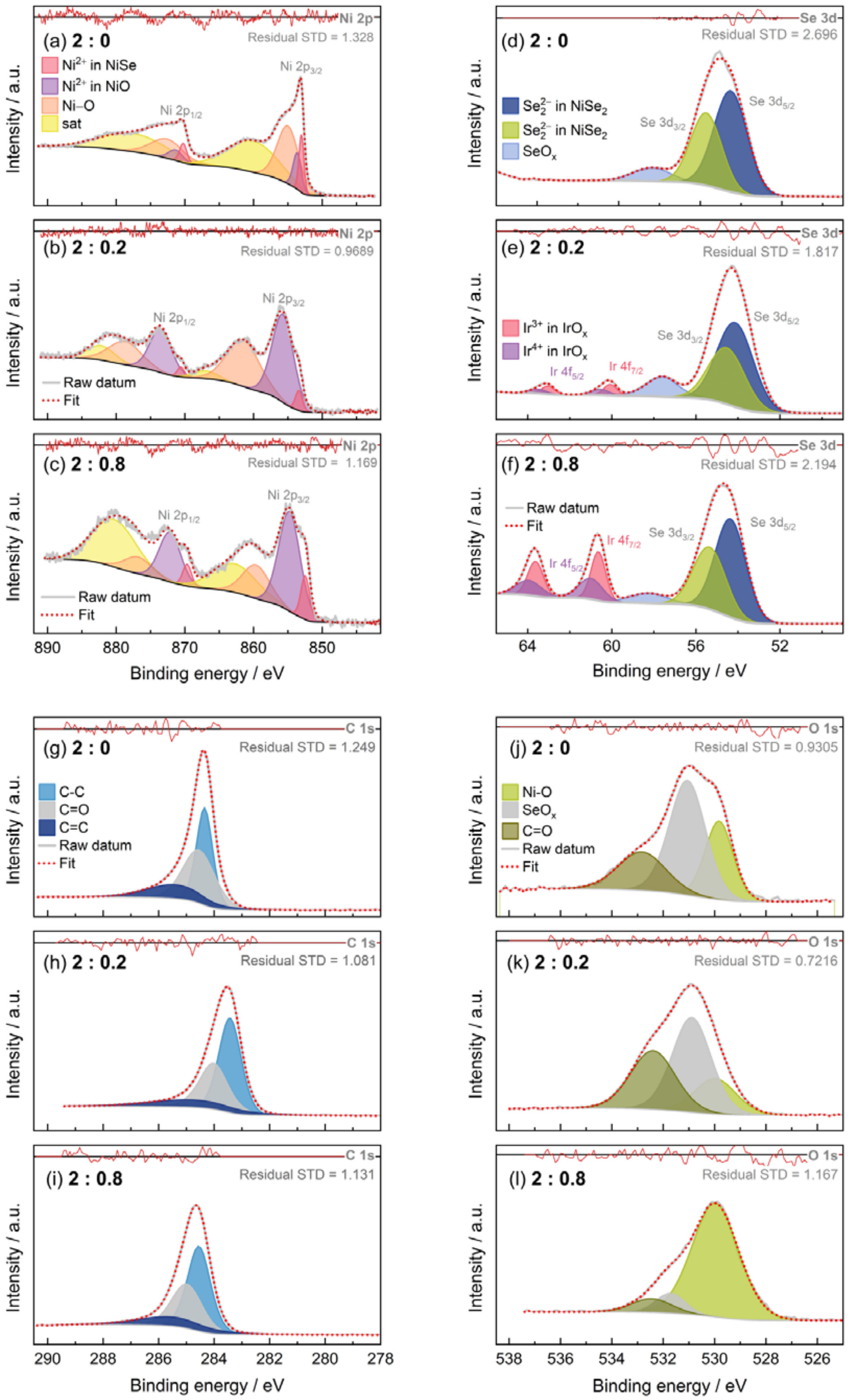

Figure S4. High-resolution XPS spectra for (a-c) Ni 2p, (d-f)Se 3d, (e-f) Ir 4f, (g-i) C 1s and (j-l) O1s core levels of pristine $NiSe_2$ and IrO_x-$NiSe_2$ with Ni:Ir molar ratios of (a, d, g, j) 2:0 ($NiSe_2$ pristine), (b, e, h, k) 2:0.2 and (c, f, i, l) 2:0.8.

For the high-resolution XPS spectra of O 1s (**Figure S4g-i**), one notice for all the samples peaks at 529.9, 531.1, and 532.4 eV assigned to the Ni-O, SeO_x, and C=O, respectively.[4] The high-resolution XPS spectra of C 1s core level feature 1s (**Figure S4j-l**), for all the samples, photoemission peaks at 284.4, 285.7, and 285.4 eV assigned to C-C, C=O, and C=C,[4] respectively, of the carbon black pearls® 2000, which was utilized as a support for the catalyst (i.e., $NiSe_2$ and IrO_x-$NiSe_2$).

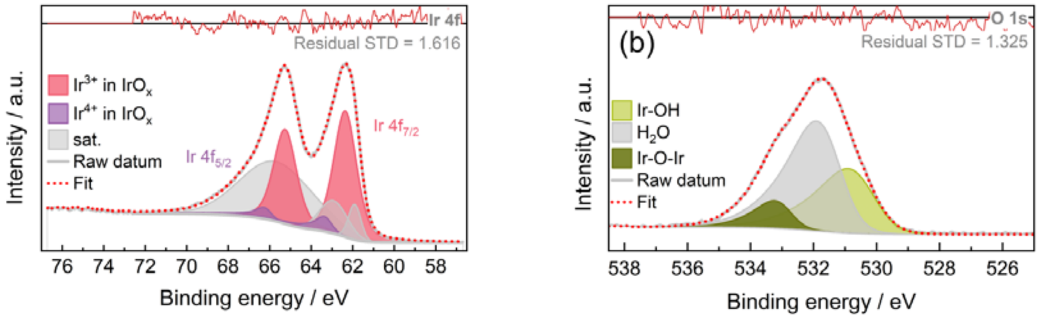

Figure S5. High-resolution XPS spectra of (a) Ir 4f and (b) O 1s core levels of pristine IrO_x.

Table S2. Fit parameters employed for modeling peaks and baseline of the XPS spectra of pristine $NiSe_2$.

Core level (component)		Pristine $NiSe_2$[a] Position eV	Area constr.	FWHM constr. eV	Position constr. eV	Line shape
Se 3d	Se 3d (A)	58.4	-	-	-	SGL(0)
	Se $3d_{5/2}$ (B)	54.5	-	-	-	SGL(0)
	Se $3d_{3/2}$ (C)	55.7	B×0.667	B×0.93	B + 1.2	SGL(0)
Ni 2p	Ni $2p_{3/2}$ (A)	853.0	-	-	-	GL(30)
	Ni $2p_{1/2}$ (B)	870.2	A×0.5	A×1.5	A + 17.2	GL(30)
	Ni $2p_{3/2}$ (C)	853.6	-	-	-	GL(30)
	Ni $2p_{1/2}$ (D)	871.3	C×0.5	C×1.7	C + 17.7	GL(30)
	Ni $2p_{3/2}$ (E)	855.0	-	-	-	GL(30)
	Ni $2p_{1/2}$ (F)	872.6	E×0.5	E×1.5	E + 17.6	GL(30)
	Ni 2p (G)	860.1	-	-	-	GL(0)
	Ni 2p (H)	877.5	-	-	-	GL(0)
O 1s	O 1s (A)	529.8	-	-	-	GL(30)
	O 1s (B)	531.1	-	-	-	GL(30)
	O 1s (C)	532.8	-	-	-	GL(30)
C 1s	C 1s (A)	284.3	-	-	-	GL(45)
	C 1s (B)	284.5	-	-	-	GL(30)
	C 1s (C)	285.4	-	-	-	GL(30)

[a]The baseline type employed was Shirley.

Table S3. Fit parameters employed for modeling peaks and baseline of the XPS spectra of pristine IrO_x.

Core level (component)		Pristine IrO_x[a]				
		Position eV	Area constr.	FWHM constr. eV	Position constr. eV	Line shape
Ir 4f	Ir $4f_{7/2}$ (A)	62.4	-	-	-	SGL(35)
	Ir $4f_{5/2}$ (B)	65.3	A×0.75	A×1	A + 2.9	SGL(35)
	Ir $4f_{7/2}$ (C)	63.4	-	-	-	DS(0.2,100)SGL(65)
	Ir $4f_{5/2}$ (D)	66.3	C×0.75	C×1	C + 2.9	DS(0.2,100)SGL(65)
	Ir 4f (E)	65.7	-	-	-	GL(0)
	Ir 4f (F)	62.9	-	-	-	GL(0)
	Ir 4f (G)	61.9	-	-	-	GL(0)
O 1s	O 1s (A)	531.6	-	-	-	LF(0.3,1.5,25,300)
	O 1s (B)	533.0	-	-	-	LF(0.3,1.5,25,300)
	O 1s (C)	530.6	-	-	-	LF(0.3,1.5,25,300)

[a]The baseline type employed was Shirley.

Table S4. Fit parameters employed for modeling peaks and baseline of the XPS spectra of IrO_x-$NiSe_2$ (Ni:Ir ratio of 2:0.2).

Core level (component)		IrO_x-$NiSe_2$ (Ni:Ir ratio of 2:0.2)[a]				
		Position eV	Area constr.	FWHM constr. eV	Position constr. eV	Line shape
Se 3d	Se 3d (A)	57.6	-	-	-	SGL(35)
	Se $3d_{5/2}$ (B)	54.1	-	-	-	SGL(0)
	Se $3d_{3/2}$ (C)	54.5	B×0.667	B×1	B + 0.4	SGL(0)
Ni 2p	Ni $2p_{3/2}$ (A)	852.5	-	-	-	GL(30)
	Ni $2p_{1/2}$ (B)	869.7	A×0.5	A×1	A + 17.2	GL(30)
	Ni $2p_{3/2}$ (C)	859.6	-	-	-	GL(30)
	Ni $2p_{1/2}$ (D)	876.8	C×0.5	C×1	C + 17.2	GL(30)
	Ni $2p_{3/2}$ (E)	854.8	-	-	-	GL(30)
	Ni $2p_{1/2}$ (F)	872.2	E×0.5	E×1	E + 17.4	GL(30)
	Ni 2p (G)	862.7	-	-	-	GL(0)
	Ni 2p (H)	880.3	-	-	-	GL(0)
Ir 4f	Ir $4f_{7/2}$ (A)	60.6	-	-	-	SGL(0)
	Ir $4f_{5/2}$ (B)	63.5	A×0.75	A×1	A + 2.95	SGL(0)
	Ir $4f_{7/2}$ (C)	60.1	-	-	-	SGL(0)
	Ir $4f_{5/2}$ (D)	63.0	C×0.75	C×1	C + 2.95	SGL(0)
O 1s	O 1s (A)	530.0	-	-	-	GL(30)
	O 1s (B)	531.7	-	-	-	GL(10)
	O 1s (C)	532.5	-	-	-	GL(10)
C 1s	C 1s (A)	283.4	-	-	-	GL(35)
	C 1s (B)	284.0	-	-	-	GL(60)
	C 1s (C)	284.7	-	-	-	GL(10)

[a]The baseline type employed was Shirley.

Table S5. Fit parameters employed for modeling peaks and baseline of the XPS spectra of IrO_x-$NiSe_2$ (Ni:Ir ratio of 2:0.8).

Core level (component)		IrO_x-$NiSe_2$ (Ni:Ir ratio of 2:0.8)[a]				
		Position eV	Area constr.	FWHM constr. eV	Position constr. eV	Line shape
Se 3d	Se 3d (A)	58.3	-	-	-	SGL(0)
	Se $3d_{5/2}$ (B)	54.4	-	-	-	SGL(0)
	Se $3d_{3/2}$ (C)	55.4	B×0.667	B×1.06	B + 1	SGL(0)
Ni 2p	Ni $2p_{3/2}$ (A)	853.4	-	-	-	GL(30)
	Ni $2p_{1/2}$ (B)	870.6	A×0.5	A×1	A + 17.2	GL(30)
	Ni $2p_{3/2}$ (C)	861.5	-	-	-	GL(30)
	Ni $2p_{1/2}$ (D)	878.7	C×0.5	C×1	C + 17.2	GL(30)
	Ni $2p_{3/2}$ (E)	855.7	-	-	-	GL(30)
	Ni $2p_{1/2}$ (F)	873.5	E×0.5	E×1	E + 17.8	GL(30)
	Ni 2p (G)	866.9	-	-	-	GL(0)
	Ni 2p (H)	882.2	-	-	-	GL(0)
Ir 4f	Ir $4f_{7/2}$ (A)	60.6	-	-	-	SGL(0)
	Ir $4f_{5/2}$ (B)	63.6	A×0.75	A×1.05	A + 2.99	SGL(0)
	Ir $4f_{7/2}$ (C)	61.0	-	-	-	SGL(0)
	Ir $4f_{5/2}$ (D)	63.9	C×0.75	C×1.05	C + 2.95	SGL(0)
O 1s	O 1s (A)	530.0	-	-	-	GL(30)
	O 1s (B)	530.9	-	-	-	GL(30)
	O 1s (C)	532.4	-	-	-	GL(30)
C 1s	C 1s (A)	284.5	-	-	-	GL(45)
	C 1s (B)	285.0	-	-	-	GL(30)
	C 1s (C)	285.5	-	-	-	GL(30)

[a]The baseline type employed was Shirley.

Table S6. XPS peaks assignment of pristine $NiSe_2$ and IrO_x, and IrO_x-$NiSe_2$ with Ni:Ir molar ratios of 2:0.2 and 2:0.8.

Core level		Binding energy / eV					
		IrO_x	$NiSe_2$	2:0.2 (Ni:Ir)	2:0.8 (Ni:Ir)	Assignment	Ref.
Se 3d	Se 3d	-	58.4	57.6	58.3	SeO_x	5
	Se $3d_{5/2}$	-	54.5	54.1	54.4	Se_2^{2-} in $NiSe_2$	5
	Se $3d_{3/2}$	-	55.7	54.5	55.4	Se_2^{2-} in $NiSe_2$	5
Ni 2p	Ni $2p_{3/2}$	-	853.0	852.5	853.4	Ni^{2+} in $NiSe_2$	5
	Ni $2p_{1/2}$	-	870.2	869.7	870.6	Ni^{2+} in $NiSe_2$	5
	Ni $2p_{3/2}$	-	853.6	859.6	861.5	Ni^{2+} in NiO	5–7
	Ni $2p_{1/2}$	-	871.3	876.8	878.7	Ni^{2+} in NiO	5–7
	Ni $2p_{3/2}$	-	855.0	854.8	855.7	Ni-O	5
	Ni $2p_{1/2}$	-	872.6	872.2	873.5	Ni-O	5
	Ni 2p	-	860.1	862.7	866.9	Satellite	5
	Ni 2p	-	877.5	880.3	882.1	Satellite	5
Ir 4f	Ir $4f_{7/2}$	62.4	-	60.1	60.6	Ir^{3+} in IrO_x	2
	Ir $4f_{5/2}$	65.3	-	63.0	63.2	Ir^{3+} in IrO_x	2
	Ir $4f_{7/2}$	63.4	-	60.6	61.0	Ir^{4+} in IrO_x	2
	Ir $4f_{5/2}$	66.3	-	63.5	63.9	Ir^{4+} in IrO_x	2
	Ir 4f	65.7	-	-	-	Satellite	8
	Ir 4f	62.9	-	-	-	Satellite	8
	Ir 4f	61.9	-	-	-	Satellite	8
O 1s	O 1s	530.6	-	530.0	530.0	Ir-O-Ir	8,9
	O 1s	531.6	-	531.7	530.9	Ir-OH	8
	O 1s	533.0	-	-	-	H_2O	8
	O 1s	-	529.8	-	-	Ni-O	10
	O 1s	-	531.1	-	-	SeO_x	10
	O 1s	-	532.8	532.5	532.4	C=O	10
C 1s	C 1s	-	284.3	283.4	284.5	C-C	10
	C 1s	-	284.5	284.0	285.0	C=O	10
	C 1s	-	285.4	284.7	285.5	C=C	10

Table S7. Atomic percentage from XPS analyses of pristine $NiSe_2$ and IrO_x-NiSe with Ni:Ir molar ratios of 2:0.2 and 2:0.4.

Catalysts	Atomic percentage (%)						
(Ni:Ir)	Ni	Se	Ir	O	C	Se/Ni*	Ir/Ni**
2 : 0 (pristine $NiSe_2$)	17.7	36.7	0	6.80	38.8	2.07	0
2: 0.2	14.5	41.7	3.1	12.2	28.6	2.88	0.21
2: 0.8	8.60	31.4	12.5	12.2	35.3	3.65	1.45

*Surface Se/Ni atomic percentage ratio from XPS.

**Surface Ir/Ni atomic percentage ratio from XPS.

S3. Structural reconstruction and OER performance

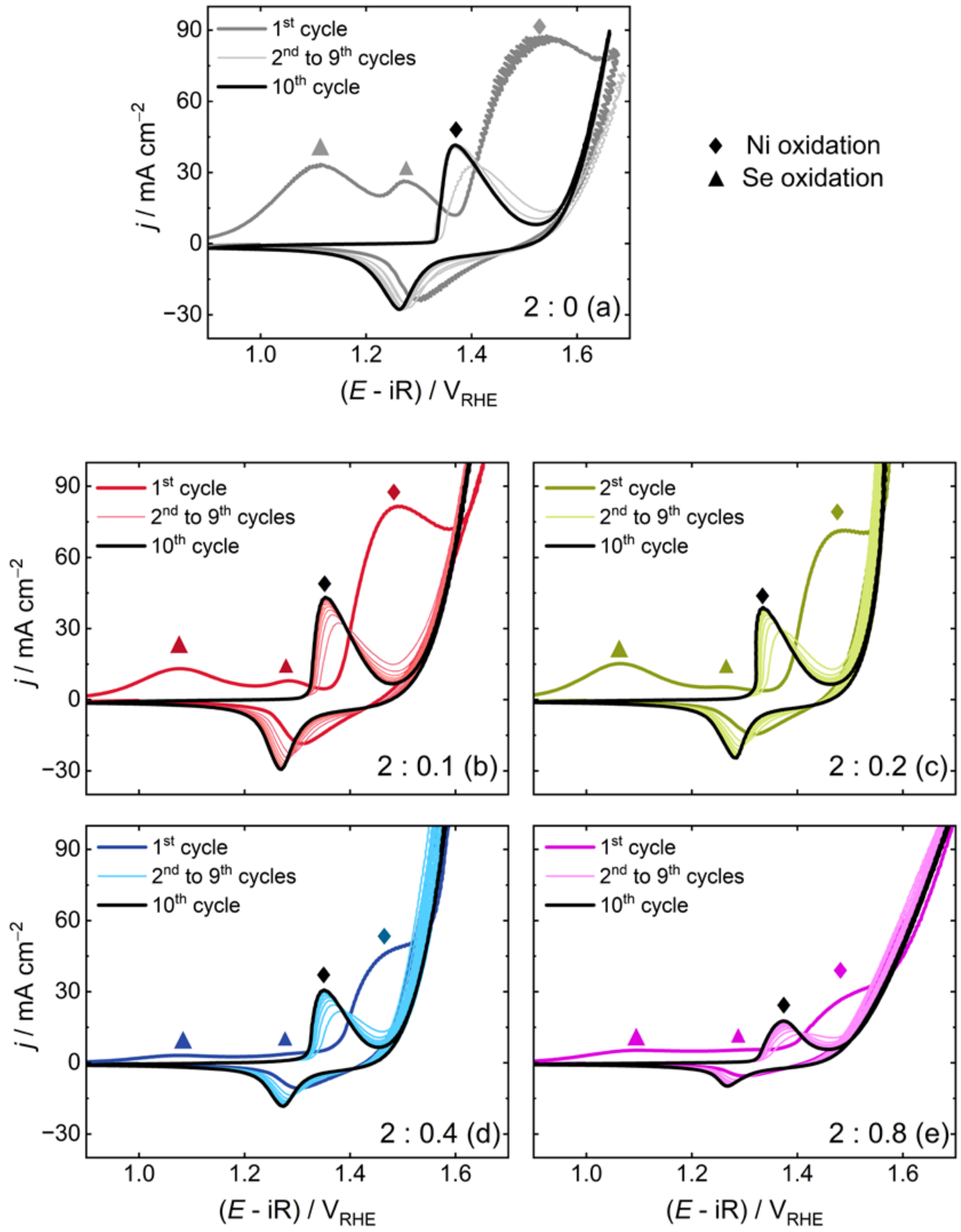

Figure S6. Structural reconstruction via cyclic voltammograms at 100 mV s^{-1} of IrO_x-$NiSe_2$ with different molar ratios of Ni:Ir with a highlight of the oxidation peaks of Se (▲) and Ni (♦). The electrolyte was an Ar-saturated solution of 1 mol L^{-1} KOH.

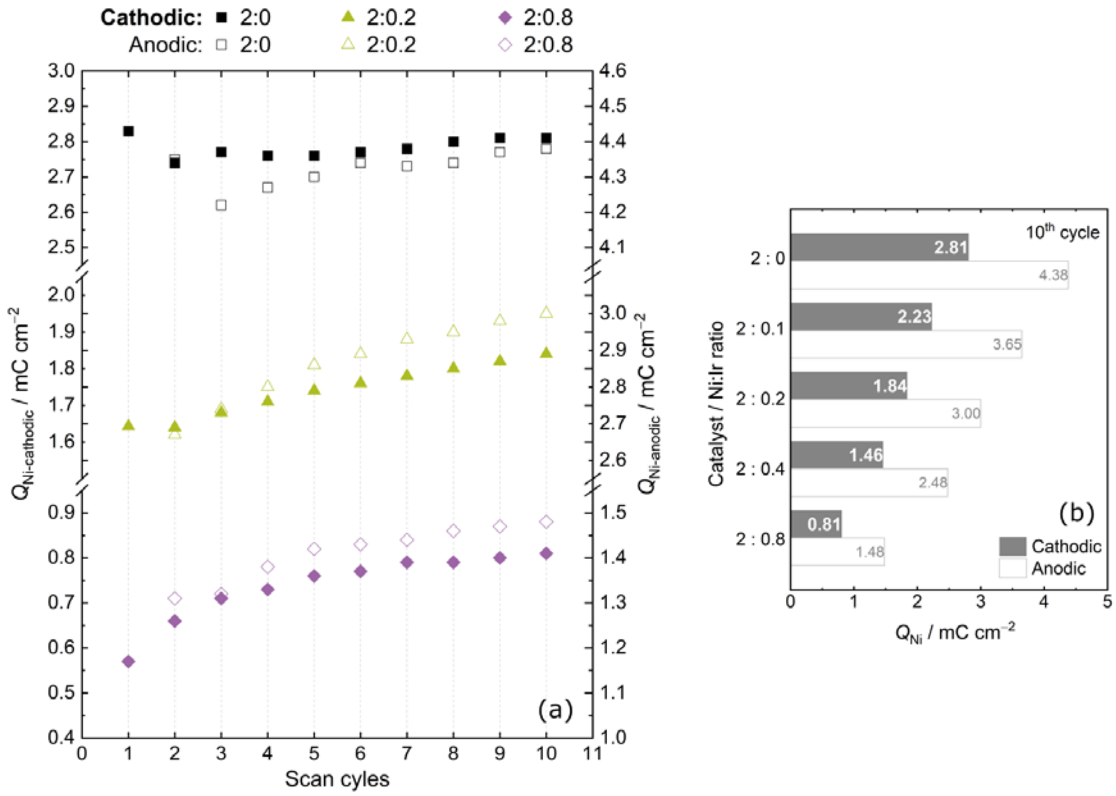

Figure S7. (a) Variation of electrochemically active Ni species during structural reconstruction via ten scans cycles at 100 mV s^{-1} of $NiSe_2$ and IrO_x-$NiSe_2$ with 2:0.2 and 2:0.8 molar ratios of Ni:Ir.* (b) Cathodic and anodic integrate charge density of nickel reduction and oxidation at tenth scan cycle for all samples.

*The anodic charge density associated with Ni^{2+} to Ni^{3+} was also integrated for comparation from the second to tenth cycle, the first anodic curve was not considered.

Table S8. Summary of electrochemical parameters for $NiSe_2$ and IrO_x-$NiSe_2$ with different molar ratios of Ni:Ir catalysts in 1 mol L^{-1} KOH.

Catalysts	E_{Ni} (V_{RHE})	E_{onset} (V_{RHE})	E_{50mA} (V_{RHE})	R_{Ω} (Ω)	η_{10} (mV_{RHE})	b (mV dec^{-1})
IrO_x	-	1.48	1.57	7.05	282	55
IrO_x-$NiSe_2$						
2 : 0	1.37	1.50	1.59	8.76	302	53
2: 0.1	1.35	1.49	1.56	6.85	291	56
2: 0.2	1.33	1.48	1.56	6.29	285	56
2: 0.4	1.35	1.47	1.57	8.13	279	61
2: 0.8	1.37	1.47	1.58	5.81	286	70

E_{Ni} is the potential of maximum value of nickel oxidation peak.

E_{onset} is the potential onset of OER obtained by tangents method (**Figure S13**).

E_{50mA} is the potential of OER at 50 mA cm^{-2}.

R_{Ω} is the ohmic resistance obtained from electrochemical impedance spectroscopy at open-circuit potential. The ohmic drop correction for the LSV and CV curves were corrected with 90% of the R_{Ω} values.

η_{10} is the overpotential (η) to achieve a current density of 10 mA cm^{-2}. The η_{10} was calculated by Equation S3:

$$\eta_{10} = E_{10\ \mathrm{mA\ cm^{-2}}} - 1.23\ \mathrm{V_{RHE}} \qquad (S3)$$

b is the Tafel slope, which was extracted from LSV measurements (Figure 4a) in the linear region of the E vs. log(j) plot.

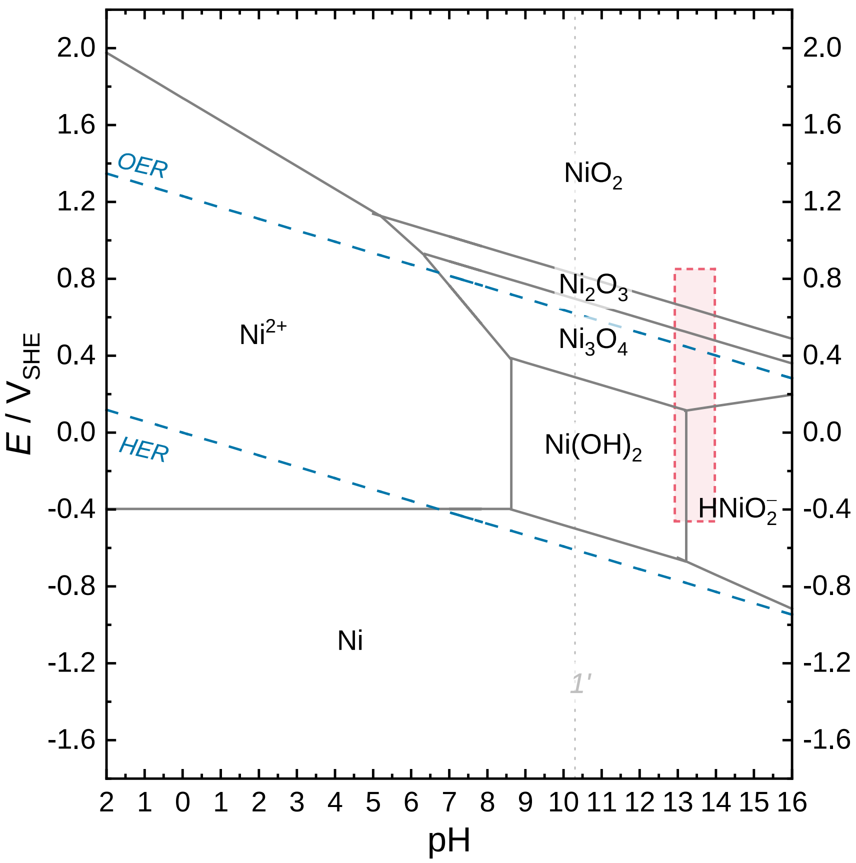

Figure S8. Nickel Pourbaix diagrams calculated from experimental thermodynamic tables[11] with aqueous ion concentrations 10^{-5} mol L^{-1} at 25 °C. The pink area highlighted indicates the potential range performed in this work (0.3 - 1.6 V_{RHE}, E_{SHE}=E_{RHE} – 0.059pH), considering a pH between 13 and 14. The light dashed line (*1'*) indicates the boundaries of the regions where the dissolved substances are relatively predominant (*1':* Ni^{2+} / $HNiO^{-}{}_{2}$, pH=10.13).

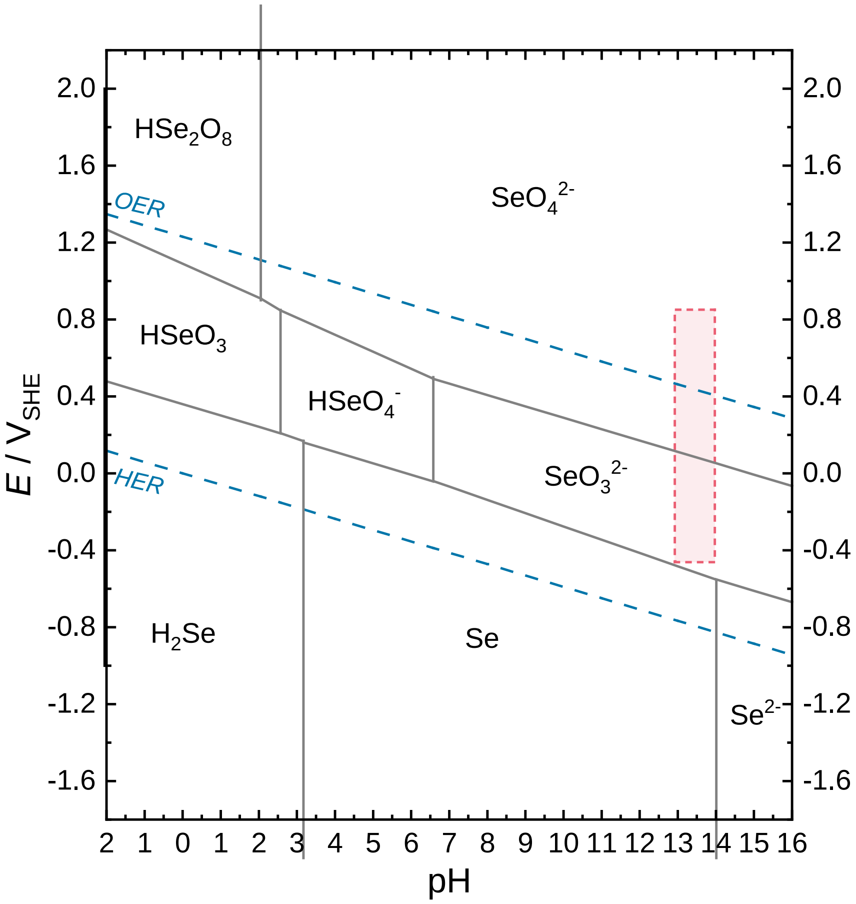

Figure S9. Selenium Pourbaix diagrams calculated from experimental thermodynamic tables[11] with aqueous ion concentrations 10^{-5} mol L^{-1} at 25 °C. The pink area highlighted indicates the potential range performed in this work (0.3 - 1.6 V_{RHE}, E_{SHE}=E_{RHE} – 0.059pH), considering a pH region between 13 and 14.

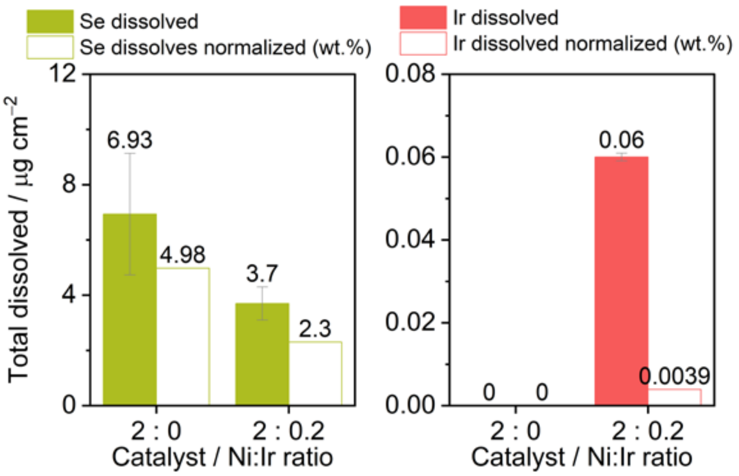

Figure S10. Total dissolution of Se and Ir during the CV protocol from 0.3–1.5 V_{RHE} at lower scan rate (2 mV s^{-1}). The white bars represent the total dissolution normalized by the weight percent (wt.%) of Se and Ir obtained by EDS analysis.

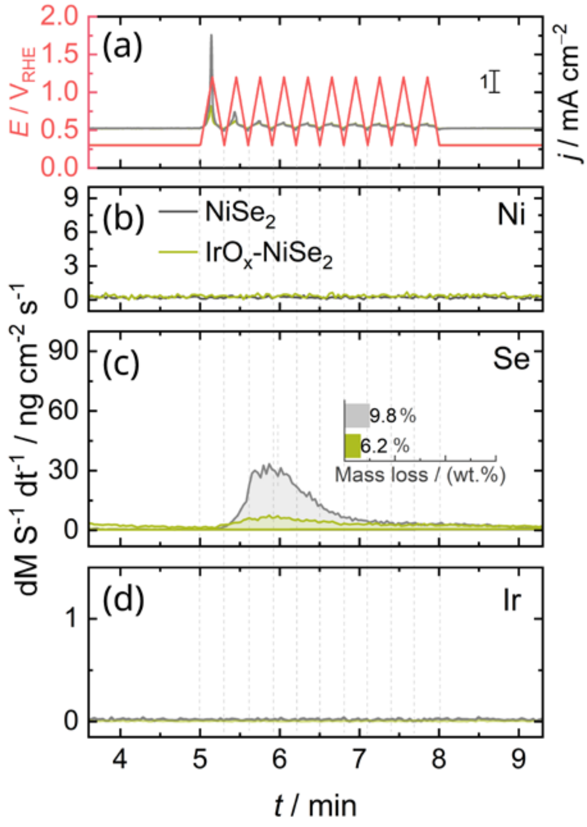

Figure S11. Dissolution of (b) Ni, (c) Se, and (d) Ir during (a) *Protocol I* (0.3–1.2 V_{RHE}) at 100 mV s^{-1}. The electrolyte was an Ar-saturated solution of 0.1 mol L^{-1} KOH.

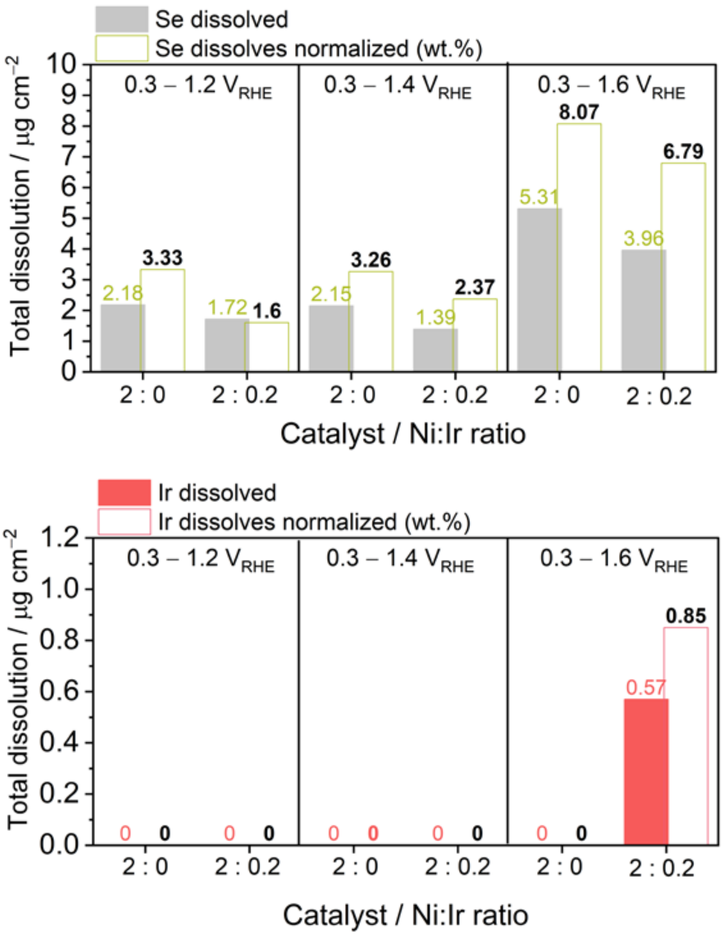

Figure S12. Total dissolution of Se and Ir during *Protocol I* (0.3–1.2 V_{RHE}), *Protocol II* (0.3–1.4 V_{RHE}) and Protocol III (0.3–1.6 V_{RHE}). The white bars represent the total dissolution normalized by the weight percent (wt.%) of Se and Ir obtained by EDS analysis.

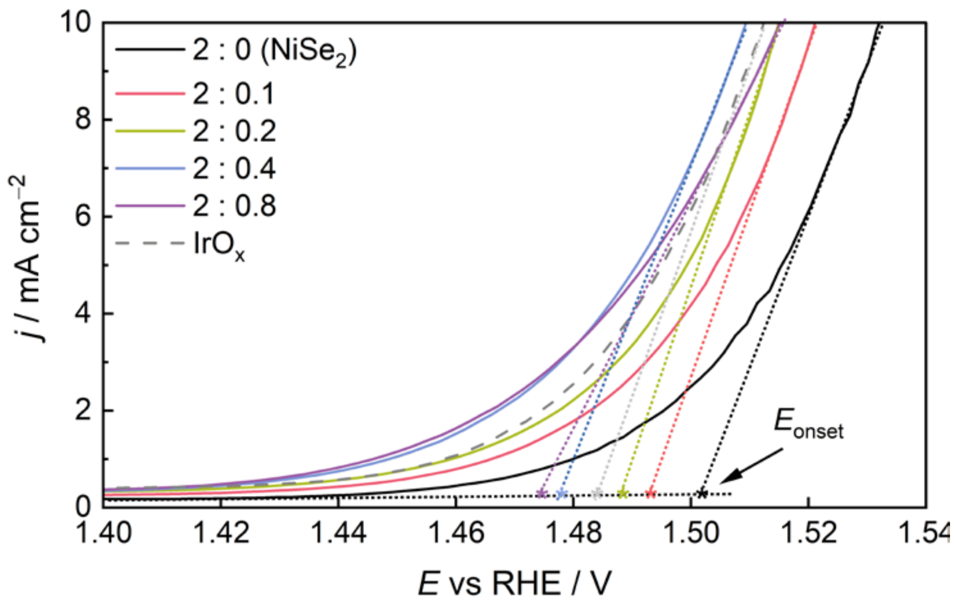

Figure S13. Determination of E_{onset} using the tangent method (current density window from 0 to 10 mA cm^{-2}) from the LSV at 5 mV s^{-1}. This method was chosen due to the non-negligible current density observed between the Ni oxidation peak (~1.35 V, Figure S6) and the onset of the OER. This residual current density is likely associated with pseudocapacitive behavior, and/or early adsorption of OER intermediates. Additionally, increased capacitive contributions may arise from the high surface area and retention of OER intermediates after surface reconstruction.

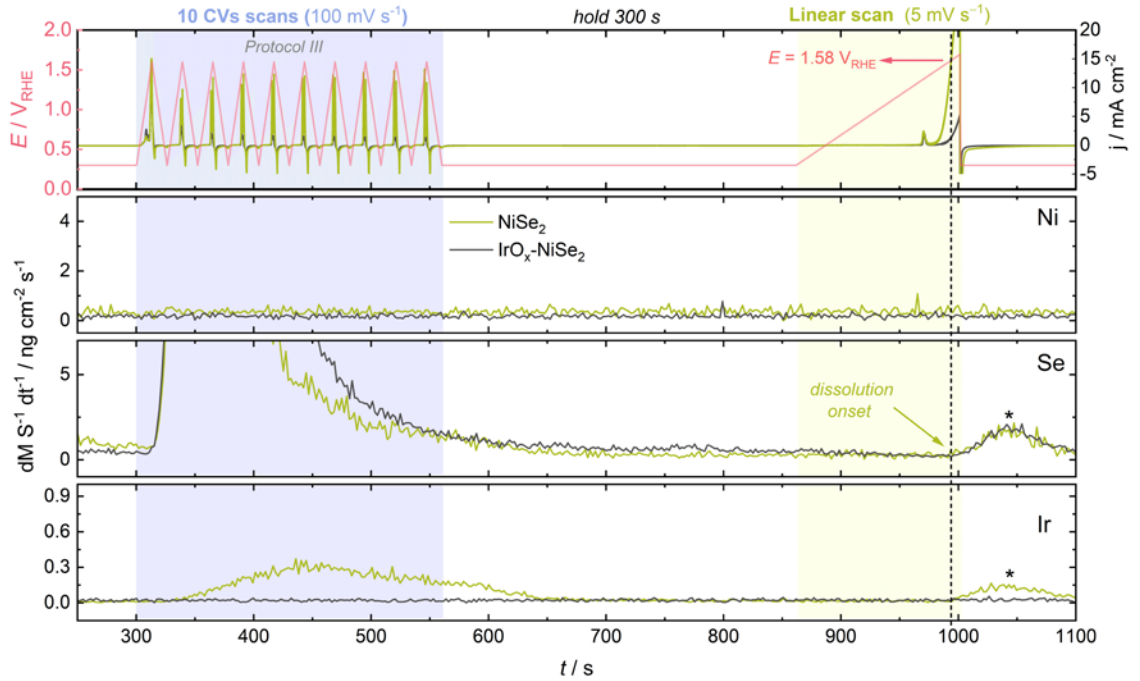

Figure S14. Dissolution of Ni, Se, and Ir during the LSV at 5 mV s^{-1} following the reconstruction process (*Protocol III*) in Ar-saturated 0.1 mol L^{-1} KOH solution.

* The dissolution of Se and Ir begins during the OER and intensifies due to the release of O_2 bubbles. This process continues even during the potential hold at 0.3 V_{RHE}, likely driven by residual O_2 on the catalyst surface, which may promote the detachment of surface species such as SeO_3^{2-} and SeO_4^{2-}.

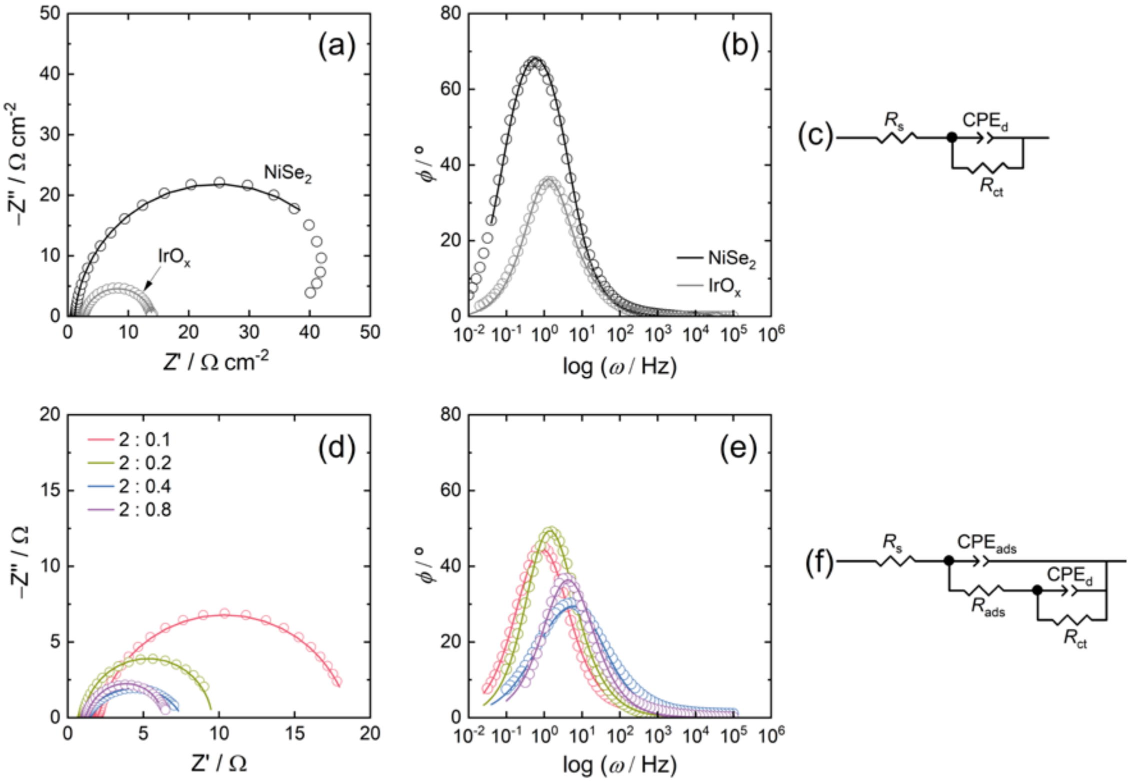

Figure S15. Complex-plane impedance and Bode diagrams at 1.47 V_{RHE} obtained after 10 CVs for (a-b) $NiSe_2$, IrO_x and (d-e) IrO_x-$NiSe_2$ with different molar ratios of Ni:Ir. The equivalent circuit used for fitting the impedance of spectra for (c) $NiSe_2$ and IrO_x, and (f) IrO_x-$NiSe_2$ with different molar ratio of Ni:Ir. The electrolyte was an Ar-saturated solution of 1 mol L^{-1} KOH.

Table S9. Resistance and capacitance density values of the electrical circuit model used to fit the impedance spectra and time constant values of the catalysts.

Components	IrO_x-$NiSe_2$ samples with different molar ratios of Ni:Ir					
	2 : 0 ($NiSe_2$)	2 : 0.1	2 : 0.2	2 : 0.4	2 : 0.8	IrO_x
R_s	1.16	1.94	0.93	1.21	1.14	2.47
$R_{ads.}$	-	0.29	0.41	0.73	0.98	-
$Q_{ads.}$	-	0.012	0.018	0.023	0.019	-
$\alpha_{f,ads.}$	-	0.89	0.92	0.85	0.91	-
$C_{ads.}$	-	0.006	0.011	0.011	0.013	-
$\tau_{ads.}$	-	0.002	0.005	0.008	0.012	-
R_{ct}	47.02	16.43	8.28	5.90	4.56	11.53
Q_d	0.039	0.029	0.031	0.036	0.015	0.032
$\alpha_{f,d}$	0.95	0.88	0.95	0.56	0.73	0.86
C_d	0.04	0.026	0.029	0.011	0.005	0.027
τ_{ct}	1.893	0.423	0.240	0.063	0.024	0.314

R_s (Ω cm^2) is the solution resistance.

R_{ct} (Ω cm^2) is the charge transfer resistance at the catalyst|electrolyte interface.

Q_d (S cm^{-2} s^{α}) is the double-layer pseudocapacitance density (also known as CPE-T) and $\alpha_{f,d}$ is the associated CPE exponent (also known as CPE-P).

C_d (F cm^{-2}) is the real double-layer capacitance density calculated from **Equation S4**.[12]

$R_{ads.}$ (Ω cm^2) is the resistance related to the adsorption of intermediate species of the OER on the catalyst surface.

$Q_{ads.}$ (S cm^{-2} s^{α}) is the pseudocapacitance density (also known as CPE-T) of the catalyst containing on its surface the adsorbed intermediate species and $\alpha_{f,ads.}$ is the associated CPE exponent (also known as CPE-P).

$C_{ads.}$ (F cm^{-2}) is the real capacitance density of the catalyst containing on its surface the adsorbed intermediate species. $C_{ads.}$ was calculated from **Equation S4**.[12]

τ_{ct} (s) is time constant associated with charge transfer time at catalyst|electrolyte interface. τ_{ct} was calculated from **Equation S5**.

$\tau_{ct,ads.}$ (s) is the time constant associated with the charge transfer time at the catalyst containing on its surface the adsorbed intermediate species. $\tau_{ct,ads.}$ was calculated from **Equation S5**.[12]

$$C = \frac{(QR)^{1/\alpha_f}}{R}, \quad (S4)$$

where R is the resistance of either R_{ct} or $R_{ads.}$, Q is the pseudocapacitance density (also known as CPE-T) of either Q_d or $Q_{ads.}$, and α_f is the CPE exponent (also known as CPE-P) of either $\alpha_{f,d}$ or $\alpha_{f,ads.}$. α_f assumes values from 0 to 1, and $\alpha_f = 1$ represents an ideal capacitor.

$$\tau = RC, \quad (S5)$$

where R is resistance of either R_{ct} or $R_{ads.}$ and C is the real capacitance density of either C_d or $C_{ads.}$.

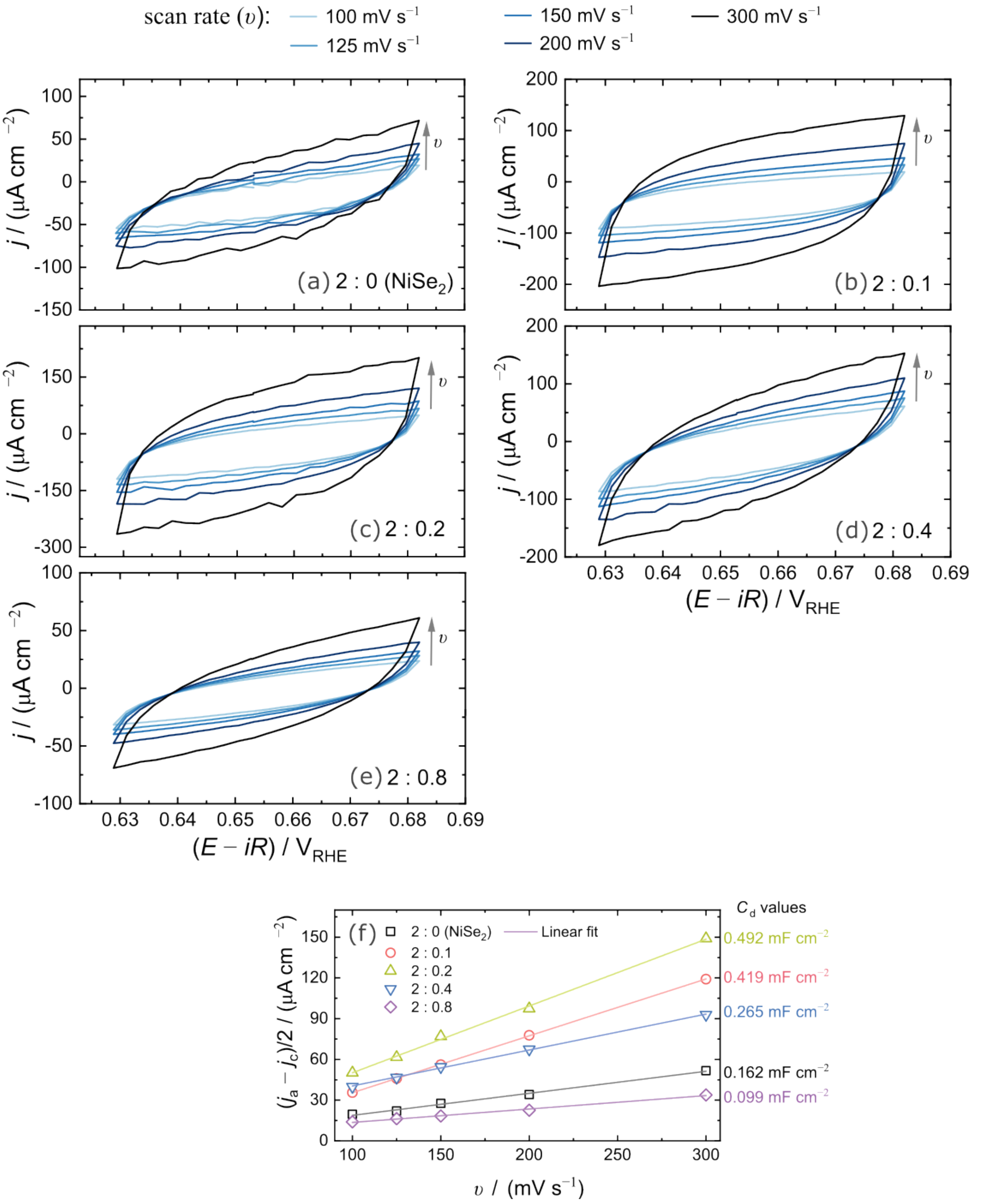

Figure S16. (a-e) Cyclic voltammograms obtained at different υ (100, 125, 150, 200, and 300 mV s^{-1}) and in a non-faradaic potential range (from 0.629 to 0.682 V_{RHE}) of $NiSe_2$ and IrO_x-$NiSe_2$ with different molar ratios of Ni:Ir. This analysis was performed without the 10 CVs (i.e., the reconstruction process). (f) $(j_a - j_c)/2$ vs. υ plots of $NiSe_2$ and IrO_x-$NiSe_2$ with different molar ratio of Ni:Ir. The electrolyte was an Ar-saturated solution of 1 mol L^{-1} KOH.

Turnover frequency (TOF)

The turnover frequency was calculated at different potentials (e.g., 1.47, 1.50, 1.53, 1.55, and 1.585 V_{RHE}) via **Equation S6**, and for that was considered 100% Faradaic efficiency for OER.[13,14]

$$\mathrm{TOF} = \frac{j}{Fzn_{\mathrm{sites}}}, \qquad (S6)$$

where j is the anodic current density at a given applied potential, F is the Faraday constant (96,485 C mol^{-1}), z is the amount of electrons in the OER (4 mol of electrons), and n_{sites} is the density of active sites of nickel-based species (cm^{-2}), which was determined by **Equation S7**: [13,14]

$$n_{\mathrm{sites}} = \frac{Q_{\mathrm{Ni}}}{Fn_{\mathrm{e}}}, \qquad (S7)$$

where n_e is the amount of electrons in the oxidation of Ni^{2+}/Ni^{3+} (1 mol of electron) and Q_{Ni} is the cathodic charge density involved with the redox process of nickel species obtained from **Figure S7b**.

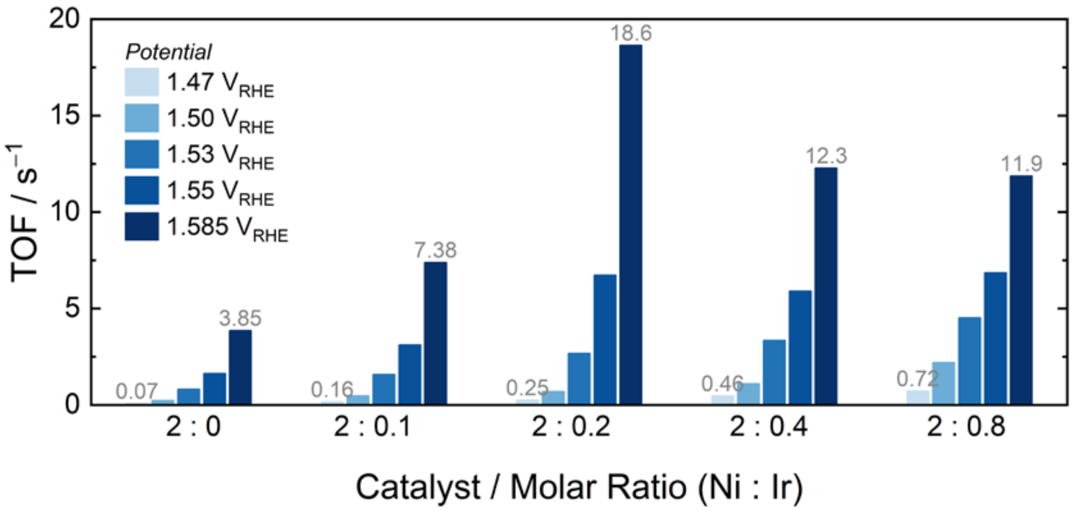

Figure S17. TOF values at different applied potentials of pristine $NiSe_2$ (Ni:Ir ratio of 2:0) and IrO_x-$NiSe_2$ with different molar ratios of Ni:Ir. The electrolyte was an Ar-saturated solution of 1 mol L^{-1} KOH.

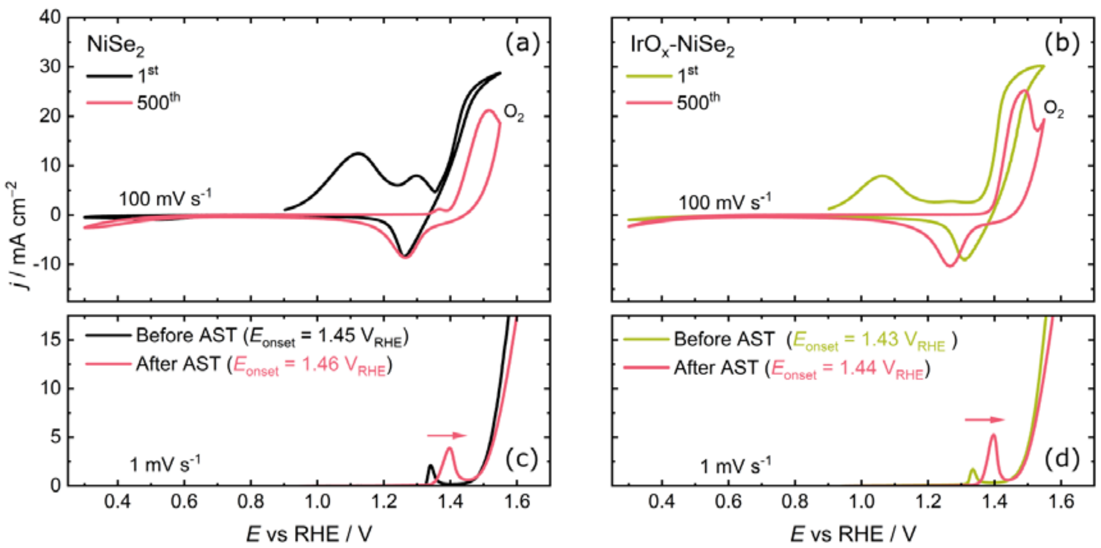

Figure S18. Accelerated stress test using a rotating disc electrode by cyclic voltammograms at 100 mV s^{-1} for (a) pristine $NiSe_2$ and (c) IrO_x-$NiSe_2$ (Ni:Ir ratio of 2:0.2) and their respective (b, d) linear sweep voltammograms at 1 mV s^{-1}. The electrolyte was an Ar-saturated solution of 1 mol L^{-1} KOH.

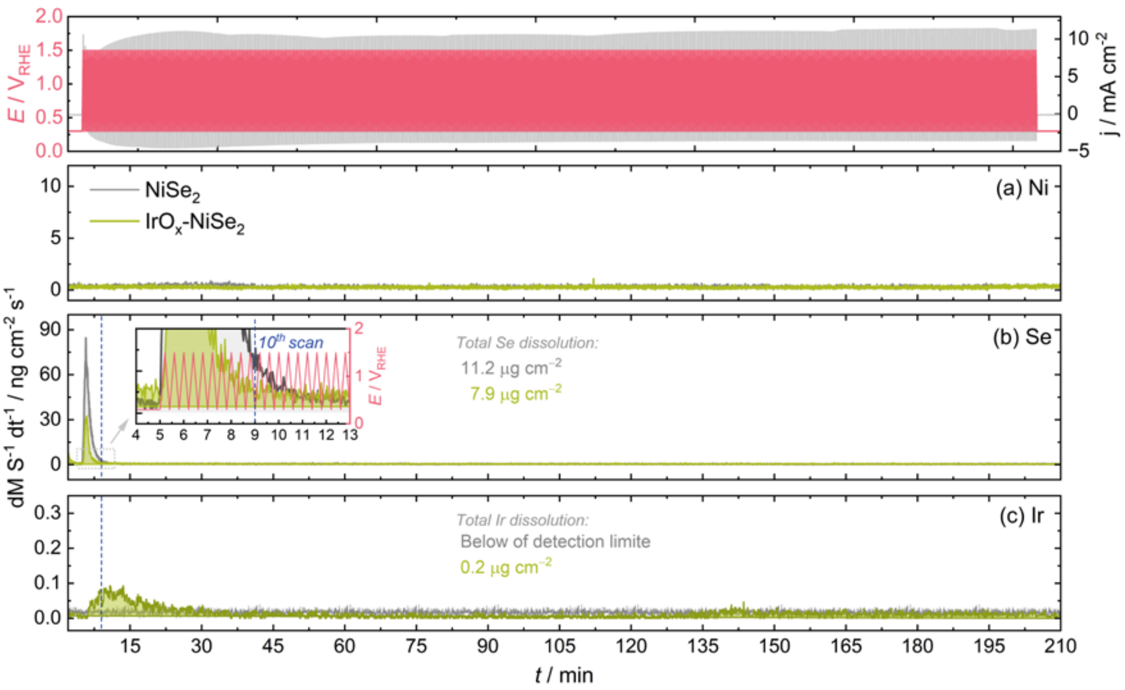

Figure S19. Dissolution of (a) Ni, (b) Se, and (c) Ir during AST performed using SFC-ICP-MS in Ar-saturated solution of 0.1 mol L^{-1} KOH.

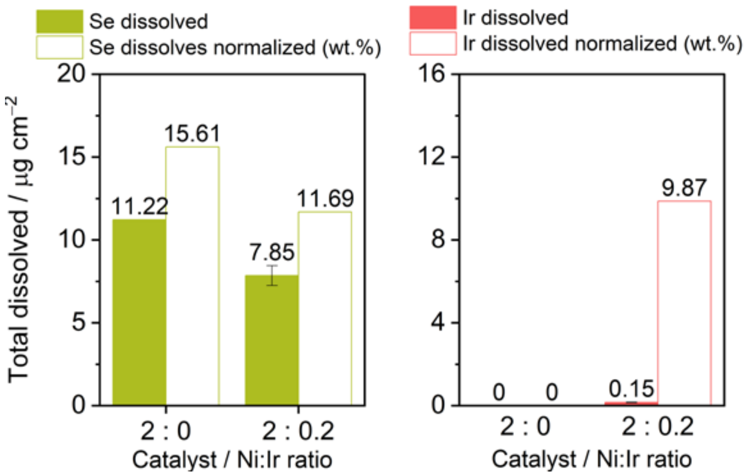

Figure S20. Total dissolution of Se and Ir during AST protocol. The white bars represent the total dissolution normalized by the weight percent (wt.%) of Se and Ir obtained by EDS analysis.

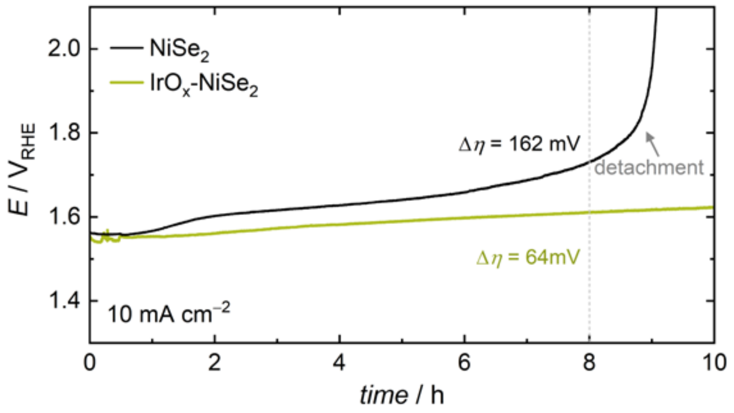

Figure S21. Chronopotentiometry curves at 10 mA cm^{-2} of pristine $NiSe_2$ and IrO_x-$NiSe_2$ (Ni:Ir ratio of 2:0.2) in a RDE setup. The electrolyte was an Ar-saturated solution of 1 mol L^{-1} KOH.

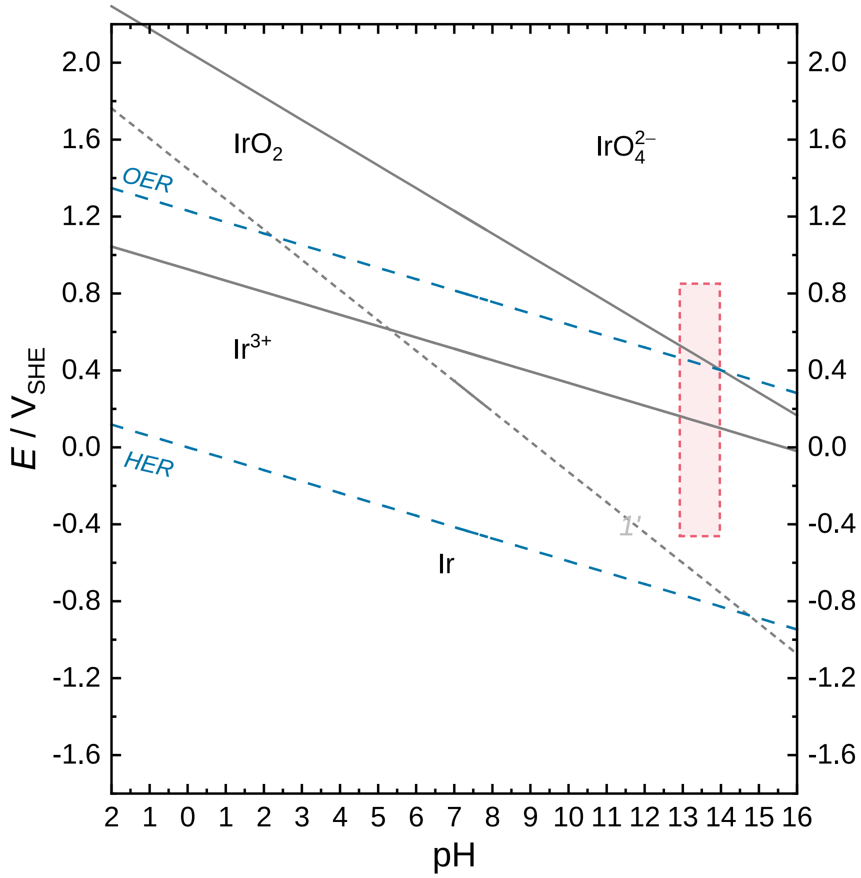

Figure S22. Iridium Pourbaix diagrams calculated from experimental thermodynamic tables[11] with aqueous ion concentrations 10^{-5} mol L^{-1} at 25 °C. The pink area highlighted indicates the potential range performed in this work (0.3 - 1.6 V_{RHE}, E_{SHE}=E_{RHE} – 0.059pH), considering a pH region between 13 and 14. The light dashed line (*1'*) indicates the boundaries of the regions where the dissolved substances are relatively predominant (*1'*: Ir^{3+} / IrO_4^{2-}, $E^{\ominus}$=1.448 – 0.1576pH).

Table S10. Comparison of OER performance of nickel-based catalyst in 1 mol L^{-1} KOH alkaline conditions.

Catalysts	OER activity		Stability assessment		Ref.
	η (mV_{RHE})	b (mV dec^{-1})	Stability approach	Outcomes	
**$NiSe_2$**	$\eta_{10} = 302$	53	CVs (500 cycles) and Chronopotentiometry (8 h) at 10 mA cm^{-2}	$\Delta\eta_{10mA}$ = 20 mV (CV) $\Delta\eta_{10mA}$ = 162 mV (CA)	***This work***
**IrO_x**	$\eta_{10} = 282$	55		-	
**IrO_x-$NiSe_2$** (Ni:Ir ratio of 2:0.2)	$\eta_{10} = 285$	56		$\Delta\eta_{10mA}$ = 14 mV (CV) $\Delta\eta_{10mA}$ = 64 mV (CA)	
$Ni(OH)_2$ 2D	$\eta_{10} = 350$	134	-	-	15
$NiSe_2$/Au	$\eta_{10} = 260$	49	Chronoamperometry (24 h) at 1.37 V_{RHE}	Unchanged	16
$NiSe_2$/NF	$\eta_{100} = 359$	128		-	15
Ni_3Se_2	$\eta_{10} = 336$	78	CVs (2000 cycles)	$\Delta\eta_{10mA}$ ~ 389 V_{RHE}	15
NiO/C	$\eta_{10} = 335$	33			16
$Ni_{0.69}Fe_{0.31}O_x$/C	$\eta_{10} = 280$	30	Chronopotentiometry (6 h) at 10 mA cm^{-2}	η_{10mA} = 280 V_{RHE} (after 6h)	17
Ir/C	$\eta_{10} = 305$	44	Chronopotentiometry (2 h)	$\Delta\eta_{10mA}$ = 194 V_{RHE}	18
$FeNiSe_2$/NF	$\eta_{100} = 293$	77	-	-	19
FeCoNiSe2	$\eta_{100} = 248$	81	Chronopotentiometry (40 h) at 10 mA cm^{-2}	Unchanged	19
$NiSe_2$/NF	-	132	CVs (500 cycles)	$\Delta\eta_{50mA}$ = 101 V_{RHE}	19
$NiSe_2$/NF	$\eta_{10} = 279$	97	Chronopotentiometry (12 h) at 10 mA cm^{-2}	E_{10mA} = 1.50 V_{RHE} (after 12h)	17
$NiSe_2$/C	-	190	Chronopotentiometry (24 h) at 10 mA cm^{-2}	$\Delta\eta_{100mA}$ = 8 V_{RHE}	17
NiSe/FTO	$\eta_{10} = 247$	76	Chronopotentiometry (10 days) at 500 mA cm^{-2}	-	20
NiS/FTO	$\eta_{10} = 310$	79	-	-	20
NiTe/FTO	$\eta_{10} = 339$	86	-	-	20
$Ni(OH)_2$	$\eta_{10} = 360$	-	-	-	20
Ir-$Ni(OH)_2$ (4%Ir)	$\eta_{10} = 230$	-	Chronopotentiometry (60 h) at 10 mA cm^{-2}	Unchanged	21
NiFe-LDH	$\eta_{10} = 280$	49	-	-	22
CoFe-LDH@NiSe	$\eta_{10} = 280$	-	-	-	23
$Ni_{45}Fe_{55}$/C	-	40	CVs (1000 cycles)	$\Delta\eta_{10mA}$ = 30 mV	24

η_{10} is the overpotential for the OER at 10 mA cm^{-2}.
b is the Tafel slope.
$\Delta\eta$ is the variation of the η ($\Delta\eta = \eta_{final} - \eta_{initial}$) at a given anodic current density. η_{final} and $\eta_{initial}$ are the overpotential for the OER at the final and initial time of chronopotentiometry curve of the stability test, respectively.
ΔE is the variation of the E ($\Delta E = E_{final} - E_{initial}$) at a given anodic current density. E_{final} and $E_{initial}$ are the potential for the OER at the final and initial time of chronopotentiometry curve of the stability test, respectively.
The term "unchanged" refers to the condition of the OER activity did not change after the stability test.

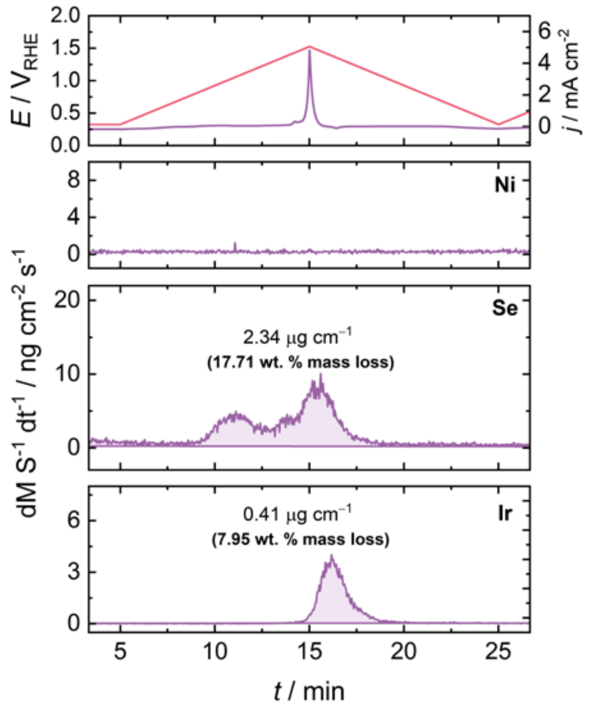

Figure S23. Dissolution of Ni, Se, and Ir for IrO_x-$NiSe_2$ with 2:0.8 ratio of Ni:Ir during the protocol at lower scan rate (2 mV s^{-1}) performed using SFC-ICP-MS in Ar-saturated solution of 0.1 mol L^{-1} KOH.

References

(1) Hussain, R. A.; Hussain, I. Fabrication and Applications of Nickel Selenide. *J Solid State Chem* 2019, *277*, 316–328. https://doi.org/10.1016/j.jssc.2019.06.015.

(2) da Silva, G. C.; Perini, N.; Ticianelli, E. A. Effect of Temperature on the Activities and Stabilities of Hydrothermally Prepared IrOx Nanocatalyst Layers for the Oxygen Evolution Reaction. *Appl Catal B* 2017, *218*, 287–297. https://doi.org/10.1016/j.apcatb.2017.06.044.

(3) Poulsen, I. A.; Garner, C. S. A Thermodynamic and Kinetic Study of Hexachloro and Aquopentachloro Complexes of Iridium(III) in Aqueous Solutions. *J Am Chem Soc* 1962, *84* (11), 2032–2037. https://doi.org/10.1021/ja00870a003.

(4) Zhang, X.; Zhang, T.; Zheng, R.; Zhao, C.; Zhou, Y.; Hu, Z. Synthesis of NiSe2 Nanoparticles Embedded into Carbon Nanoplate as Lithium/Sodium Ion Battery Anodes. *Int J Electrochem Sci* 2021, *16* (12), 211210. https://doi.org/10.20964/2021.12.17.

(5) Nayak, A. K.; Pradhan, D. NiSe 2 -Nanooctahedron as an Efficient Electrocatalyst for Overall Water Splitting. *ACS Appl Energy Mater* 2025, *8* (4), 2088–2102. https://doi.org/10.1021/acsaem.4c02585.

(6) Zheng, X.; Han, X.; Liu, H.; Chen, J.; Fu, D.; Wang, J.; Zhong, C.; Deng, Y.; Hu, W. Controllable Synthesis of Ni *x* Se ($0.5 \le x \le 1$) Nanocrystals for Efficient Rechargeable Zinc–Air Batteries and Water Splitting. *ACS Appl Mater Interfaces* 2018, *10* (16), 13675–13684. https://doi.org/10.1021/acsami.8b01651.

(7) Kareem, A.; Jose, E.; Thenmozhi, K.; Senthilkumar, S. In Situ Growth of Trimetallic FeCoNi Selenide Nanosheets on Nickel Foam for Electrocatalytic Water Splitting. *ACS Appl Nano Mater* 2025, *8* (16), 8424–8432. https://doi.org/10.1021/acsanm.5c01097.

(8) Ding, L.; Li, K.; Wang, W.; Xie, Z.; Yu, S.; Yu, H.; Cullen, D. A.; Keane, A.; Ayers, K.; Capuano, C. B.; Liu, F.; Gao, P.-X.; Zhang, F.-Y. Amorphous Iridium Oxide-Integrated Anode Electrodes with Ultrahigh Material Utilization for Hydrogen Production at Industrial Current Densities. *Nanomicro Lett* 2024, *16* (1), 203. https://doi.org/10.1007/s40820-024-01411-7.

(9) Foster, J.; Lyu, X.; Serov, A.; Mauger, S.; Padgett, E.; Pylypenko, S. X-Ray Photoelectron Spectroscopy Investigation of Iridium Oxide Catalyst Layers: Insights into the Catalyst-Ionomer Interface. *Electrochim Acta* 2025, *517*, 145705. https://doi.org/10.1016/j.electacta.2025.145705.

(10) Zheng, J.; Gong, Q.; Cheng, X.; Gong, S.; Yang, W.; Huang, L. Microwave Synthesis of Carbon-Supported Cobalt Nickel Selenide Ternary Catalyst Toward the Oxygen Reduction Reaction. *ChemElectroChem* 2018, *5* (14), 2019–2028. https://doi.org/10.1002/celc.201800190.

(11) Pourbaix, M. Atlas of Electrochemical Equilibria in Aqueous Solutions. *National Association of Corrosion Engineers* 1974.

(12) Yang, W.; Moehl, T.; Service, E.; Tilley, S. D. Operando Analysis of Semiconductor Junctions in Multi-Layered Photocathodes for Solar Water Splitting by Impedance Spectroscopy. *Adv Energy Mater* 2021, *11* (9), 2003569. https://doi.org/10.1002/aenm.202003569.

(13) Qian, H.; Wei, J.; Yu, C.; Tang, F.; Jiang, W.; Xia, D.; Gan, L. In Situ Quantification of the Active Sites, Turnover Frequency, and Stability of Ni–Fe (Oxy)Hydroxides for the Oxygen Evolution Reaction. *ACS Catal* 2022, *12* (22), 14280–14289. https://doi.org/10.1021/ACSCATAL.2C03898.

(14) Anantharaj, S.; Karthik, P. E.; Noda, S. The Significance of Properly Reporting Turnover Frequency in Electrocatalysis Research. *Angewandte Chemie International Edition* 2021, *60* (43), 23051–23067. https://doi.org/10.1002/anie.202110352.

(15) Wu, H.; Lu, X.; Zheng, G.; Ho, G. W. Topotactic Engineering of Ultrathin 2D Nonlayered Nickel Selenides for Full Water Electrolysis. *Adv Energy Mater* 2018, *8* (14). https://doi.org/10.1002/aenm.201702704.

(16) Swesi, A. T.; Masud, J.; Liyanage, W. P. R.; Umapathi, S.; Bohannan, E.; Medvedeva, J.; Nath, M. Textured NiSe2 Film: Bifunctional Electrocatalyst for Full Water Splitting at Remarkably Low Overpotential with High Energy Efficiency. *Sci Rep* 2017, *7* (1), 2401. https://doi.org/10.1038/s41598-017-02285-z.

(17) Kareem, A.; Jose, E.; Thenmozhi, K.; Senthilkumar, S. In Situ Growth of Trimetallic FeCoNi Selenide Nanosheets on Nickel Foam for Electrocatalytic Water Splitting. *ACS Appl Nano Mater* 2025, *8* (16), 8424–8432. https://doi.org/10.1021/acsanm.5c01097.

(18) Chen, S.; Mi, J.-L.; Zhang, P.; Feng, Y.-H.; Yong, Y.-C.; Shi, W.-D. Control Synthesis of Nickel Selenides and Their Multiwalled Carbon Nanotubes Composites as Electrocatalysts for Enhanced Water Oxidation. *The Journal of Physical Chemistry C* 2018, *122* (45), 26096–26104. https://doi.org/10.1021/acs.jpcc.8b09259.

(19) Qiu, Y.; Xin, L.; Li, W. Electrocatalytic Oxygen Evolution over Supported Small Amorphous Ni–Fe Nanoparticles in Alkaline Electrolyte. *Langmuir* 2014, *30* (26), 7893–7901. https://doi.org/10.1021/la501246e.

(20) Dasgupta, B.; Yao, S.; Mondal, I.; Mebs, S.; Schmidt, J.; Laun, K.; Zebger, I.; Dau, H.; Driess, M.; Menezes, P. W. A Knowledge-Based Molecular Single-Source Precursor Approach to Nickel Chalcogenide Precatalysts for Electrocatalytic Water, Alcohol, and Aldehyde Oxidations. *ACS Nano* 2024, *18* (50), 33964–33976. https://doi.org/10.1021/acsnano.4c08058.

(21) Xing, Y.; Ku, J.; Fu, W.; Wang, L.; Chen, H. Inductive Effect between Atomically Dispersed Iridium and Transition-Metal Hydroxide Nanosheets Enables Highly Efficient Oxygen Evolution Reaction. *Chemical Engineering Journal* 2020, *395*, 125149. https://doi.org/10.1016/j.cej.2020.125149.

(22) Yu, L.; Yang, J. F.; Guan, B. Y.; Lu, Y.; Lou, X. W. (David). Hierarchical Hollow Nanoprisms Based on Ultrathin Ni-Fe Layered Double Hydroxide Nanosheets with Enhanced Electrocatalytic Activity towards Oxygen Evolution. *Angewandte Chemie International Edition* 2018, *57* (1), 172–176. https://doi.org/10.1002/anie.201710877.

(23) Jeghan, S. M. N.; Kim, D.; Lee, Y.; Kim, M.; Lee, G. Designing a Smart Heterojunction Coupling of Cobalt-Iron Layered Double Hydroxide on Nickel Selenide Nanosheets for Highly Efficient Overall Water Splitting Kinetics. *Appl Catal B* 2022, *308*, 121221. https://doi.org/10.1016/j.apcatb.2022.121221.

(24) Görlin, M.; Ferreira de Araújo, J.; Schmies, H.; Bernsmeier, D.; Dresp, S.; Gliech, M.; Jusys, Z.; Chernev, P.; Kraehnert, R.; Dau, H.; Strasser, P. Tracking Catalyst Redox States and Reaction Dynamics in Ni–Fe Oxyhydroxide Oxygen Evolution Reaction Electrocatalysts: The Role of Catalyst Support and Electrolyte PH. *J Am Chem Soc* 2017, *139* (5), 2070–2082. https://doi.org/10.1021/jacs.6b12250.
